# Bottom-Up Assembly of Synthetic Cells with a DNA Cytoskeleton

**DOI:** 10.1021/acsnano.1c10703

**Published:** 2022-04-04

**Authors:** Kevin Jahnke, Vanessa Huth, Ulrike Mersdorf, Na Liu, Kerstin Göpfrich

**Affiliations:** † Biophysical Engineering Group, Max Planck Institute for Medical Research, Jahnstraße 29, D-69120 Heidelberg, Germany; ‡ Department of Physics and Astronomy, Heidelberg University, D-69120 Heidelberg, Germany; § Department of Biomolecular Mechanisms, Max Planck Institute for Medical Research, Jahnstraße 29, D-69120 Heidelberg, Germany; ⊥ 2nd Physics Institute, University of Stuttgart, Im Pfaffenwaldring 57, D-70569 Stuttgart, Germany; ∥ Max Planck Institute for Solid State Research, Heisenbergstraße 1, D-70569 Stuttgart, Germany

**Keywords:** DNA nanotechnology, giant unilamellar vesicles, azobenzene, DNA nanotube, synthetic cell, bottom-up synthetic biology

## Abstract

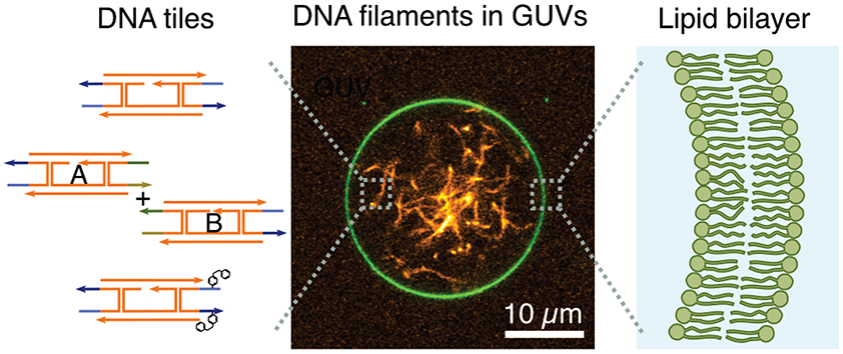

Cytoskeletal elements, like actin and myosin, have been reconstituted inside lipid vesicles toward the vision to reconstruct cells from the bottom up. Here, we realize the de novo assembly of entirely artificial DNA-based cytoskeletons with programmed multifunctionality inside synthetic cells. Giant unilamellar lipid vesicles (GUVs) serve as cell-like compartments, in which the DNA cytoskeletons are repeatedly and reversibly assembled and disassembled with light using the *cis*–*trans* isomerization of an azobenzene moiety positioned in the DNA tiles. Importantly, we induced ordered bundling of hundreds of DNA filaments into more rigid structures with molecular crowders. We quantify and tune the persistence length of the bundled filaments to achieve the formation of ring-like cortical structures inside GUVs, resembling actin rings that form during cell division. Additionally, we show that DNA filaments can be programmably linked to the compartment periphery using cholesterol-tagged DNA as a linker. The linker concentration determines the degree of the cortex-like network formation, and we demonstrate that the DNA cortex-like network can deform GUVs from within. All in all, this showcases the potential of DNA nanotechnology to mimic the diverse functions of a cytoskeleton in synthetic cells.

Growth and development, organization, adaptation, stimuli response, or reproduction—many of the features that characterize living cells—are dependent on their active cytoskeletons. Engineering multifunctional cytoskeletons for synthetic cells thus brings us closer toward the audacious vision of engineering life from the bottom up. The reconstitution of natural cytoskeletal filaments, like actin or microtubules, inside cell-sized lipid vesicles sheds light on the minimal set of proteins needed for the formation of biologically relevant structures, such as actin rings^1−3^ or membrane protrusions.^4−7^ Concomitantly, the combination of these minimal functional units proved to be challenging because the functionality of one element is often compromised by the addition of others. This hints that a true engineering approach to synthetic biology may benefit from customized materials to not only mimic but also ultimately exceed the functionality of natural cytoskeletons.

Here, DNA nanotechnology allows nanoscale objects that self-assemble into predefined architectures, including transmembrane channels,^8−11^ motors,^12−14^ scaffolds,^15−17^ and, in particular, DNA filaments, to be precisely and programmably designed.^18−21^ Despite their obvious relevance for bottom-up synthetic biology, most of these components have not yet been reconstituted inside lipid vesicles. This is, in particular, desirable in the case of DNA filaments, as features like the formation of ring-like structures or protrusions require confinement. While the encapsulation of DNA scaffolds into lipid vesicles to provide passive mechanical support has been achieved,^15^ it is now crucial to engineer multiple DNA-based dynamic functions inside GUVs. Toward this aim, DNA filaments as more versatile cytoskeleton mimics have only very recently been encapsulated into water-in-oil droplets.^22^ However, the high surface tension compared to that in lipid vesicles and the lack of a surrounding aqueous environment prevents the implementation of downstream functions.

Here, we realize a programmable and multifunctional DNA cytoskeleton composed of DNA filaments. The DNA filaments can be engineered to self-assemble reversibly upon a light stimulus inside giant unilamellar lipid vesicles (GUVs). Moreover, DNA filaments can be bundled using molecular crowders, and their persistence length is tuned via the choice of crowder. Finally, they can be engineered to form ring-like architectures and membrane protrusions in confinement.

## Results and Discussion

### Assembly and Encapsulation of DNA Cytoskeletons into GUVs

**Figure 1:**
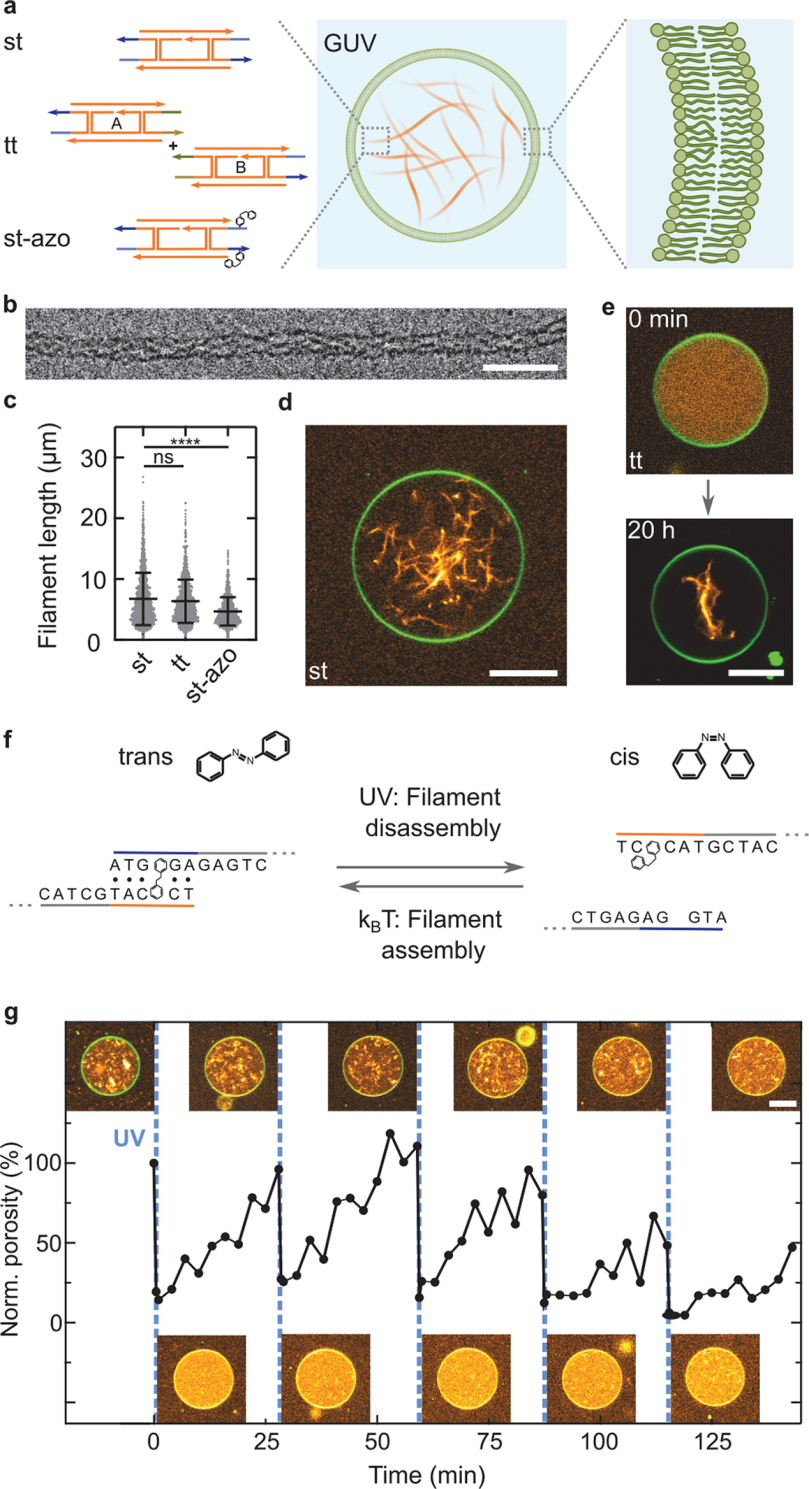
DNA cytoskeletons can be assembled reversibly inside GUVs as lipid-bilayer-enclosed synthetic cell models. (a) Schematic representation of a GUV containing a DNA cytoskeleton composed of DNA filaments. DNA cytoskeletons were assembled from a single tile (st) with sticky overhangs, two tiles (tt) with orthogonal complementarity, or single tiles modified internally with two azobenzene moieties (st-azo). The asterisk indicates the position of a single-stranded overhang modified with a fluorophore. (b) Cryo-electron micrograph of an st DNA filament. Scale bar: 50 nm. (c) DNA filament length (*n* > 1000 filaments, mean ± SD). The st and tt filaments have the same length (*p* = 0.16), and st-azo filaments are shorter (*p* ≤ 0.001). (d) Confocal image of an st DNA cytoskeleton (orange, labeled with Cy3, λ_ex_ = 561 nm) inside a GUV (green, 69% DOPC, 30% DOPG, 1% Atto488-DOPE, λ_ex_ = 488 nm). Scale bar: 10 μm. (e) Confocal images of tt cytoskeletons prior to (0 h) and after assembly (20 h) inside a GUV. Scale bar: 10 μm. (f) Schematic representation of the st overhang modified with azobenzene (st-azo) for reversible cytoskeleton assembly with UV light. (g) Light-mediated reversible assembly of 500 nM st-azo cytoskeletons inside a GUV. The porosity measures the degree of filament polymerization over time. Time points of UV illumination (15 s) are indicated (blue dashed line). Insets depict confocal images of the same GUV at the respective time points. Scale bar: 10 μm.

First, we set out to reconstitute DNA cytoskeletons inside GUVs (Figure 1a). The DNA cytoskeleton is assembled from individual DNA tiles composed of five single-stranded DNA oligomers that self-assemble into hollow filamentous DNA nanotubes.^18^ To realize versatile functions inside GUVs, we use three different sets of DNA tiles for the filament formation: The single-tile (st) DNA filaments consist of only one type of DNA tile with sequence complementary five nucleotide long sticky overhangs on its ends. The two-tile (tt) design uses two orthogonal tiles, which can only polymerize into filaments once they are combined. Here, the sticky overhang of tile A is designed to bind to tile B but not to itself. Alternatively, the st DNA tiles are modified with light-sensitive azobenzene moieties at the sticky overhangs of the single tile (st-azo). We verify the assembly of all three types of tiles into DNA filaments with cryo-electron microscopy (Figure 1b and Figure S1), revealing a diameter of 14.5 ± 1.8 nm consistent with the formation of a 12–14 helix bundle for all tile designs (Figure S2). Furthermore, we analyze the filament length with confocal microscopy, revealing that st and tt DNA filaments do not differ significantly in their mean length of 6.8 ± 4.3 and 6.4 ± 3.6 μm, respectively (Figure 1c). On the other hand, st-azo filaments are shorter with a mean length of 4.7 ± 2.3 μm, due to the addition of azobenzene into the sticky overhangs. Note that there is likely always a small fraction of azobenzenes in the *cis* state, which limits filament growth. Importantly, micrometer-long filaments are successfully formed from all three types of tiles. It is notable that st DNA filaments assemble inside GUVs in a high yield (Figure 1d). This is achieved by first encapsulating the DNA tiles together with small unilamellar lipid vesicles (SUVs, consisting of 69% DOPC, 30% DOPG, 1% Atto488-DOPE) inside surfactant-stabilized water-in-oil droplets. In the presence of negatively charged surfactants and divalent ions, the SUVs fuse at the droplet periphery to form a spherical supported lipid bilayer at the water–oil interface.^23−25^ By breaking up the water-in-oil emulsion with a destabilizing surfactant, we are able to release free-standing DNA-tile-containing GUVs into the aqueous phase (Figure S3). Importantly, the DNA filaments assemble in confinement. They only form after the release into the aqueous phase due to DNA filament disassembly in the presence of negatively charged surfactants in the oil phase (Figure S4). After the GUV release and DNA filament assembly, st DNA filaments are homogeneously distributed and dynamic in the lumen of GUVs (Movie S1). We find that both st and tt tiles form filaments inside GUVs; however, the assembly kinetics are slower for the tt tiles (Figure 1e; see Figure S5 for more examples). By quantifying the assembly processes inside GUVs for st and tt DNA filaments, we observe that st DNA filament assembly takes about 30 min and is at least 2-fold faster than tt filament assembly (Figure S6). After longer periods of time (20 h), the tt filaments cluster due to the presence of Mg^2+^ (Figure S7).

### Light-Triggered Reversible Assembly of DNA Cytoskeletons

An important feature of cellular cytoskeletons is the possibility to reversibly assemble inside cells in a stimuli-responsive manner.^26,27^ Here, we exploit the technological advantage of DNA nanotechnology to gain full spatiotemporal control over the assembly and disassembly of the DNA filaments inside GUVs. For this purpose, we place a photoswitchable azobenzene moiety internally in the sticky overhangs of the st design. In its *trans* form, azobenzene can intercalate into DNA and induce base-stacking interactions that stabilize the DNA duplex (Figure 1f).^28^ However, in its *cis* form, azobzenze blocks the hydrogen bonds of its neighboring base. We position the azobenzene moiety two bases before the end of the five nucleotide long sticky overhang, such that the *trans*–*cis* isomerization should render the connection between the tiles unstable and hence induce filament disassembly (Figure S8). Importantly, filament disassembly can be triggered locally with UV illumination. Over time, azobenzene relaxes back into the energetically favorable *trans* form, which, in turn, allows the filaments composed of the st-azo tiles to reassemble.

We encapsulate the st-azo tiles into GUVs and follow the assembly and dissassembly process inside individual GUVs with confocal microscopy. The *trans*-to-*cis* isomerization and thereby filament disassembly was induced with 15 s of illumination with a UV lamp integrated into the confocal microscope (Figure 1g and Figure S9). We quantify the reversibility by analyzing the normalized porosity of the DNA filament fluorescence, which serves as a measure for the degree of polymerization. Note that the porosity drops within seconds from 100 to 18.2% after UV illumination. We verify that standard st DNA filaments without azobenzene do not disassemble after UV illumination (Figures S10 and S11). Over the course of 30 min, the azobenzene relaxes back into its *trans*-isomer, leading to filament reassembly inside the same GUV at a rate comparable to that of the tt filaments (Figure S6). The initial porosity is nearly restored (96.0%, Figure S12). The full disassembly–assembly cycle can be repeated reproducibly five times with some fatigue in the last two cycles. The imperfect reassembly may likely be attributed to Mg^2+^-mediated clustering of DNA filaments, which becomes apparent at longer time scales (>1 h; for additional examples, see Figure S13). Note that, due to the short illumination times of seconds, UV damage is expected to be minimal even after repeated cycles.^29^

We have shown that DNA filaments can be reconstituted into GUVs and that DNA nanotechnology allows for the implementation of the highly dynamic assembly and disassembly with full spatiotemporal control inside the confinement of a synthetic cell.

### Bundling of DNA Filaments

**Figure 2:**
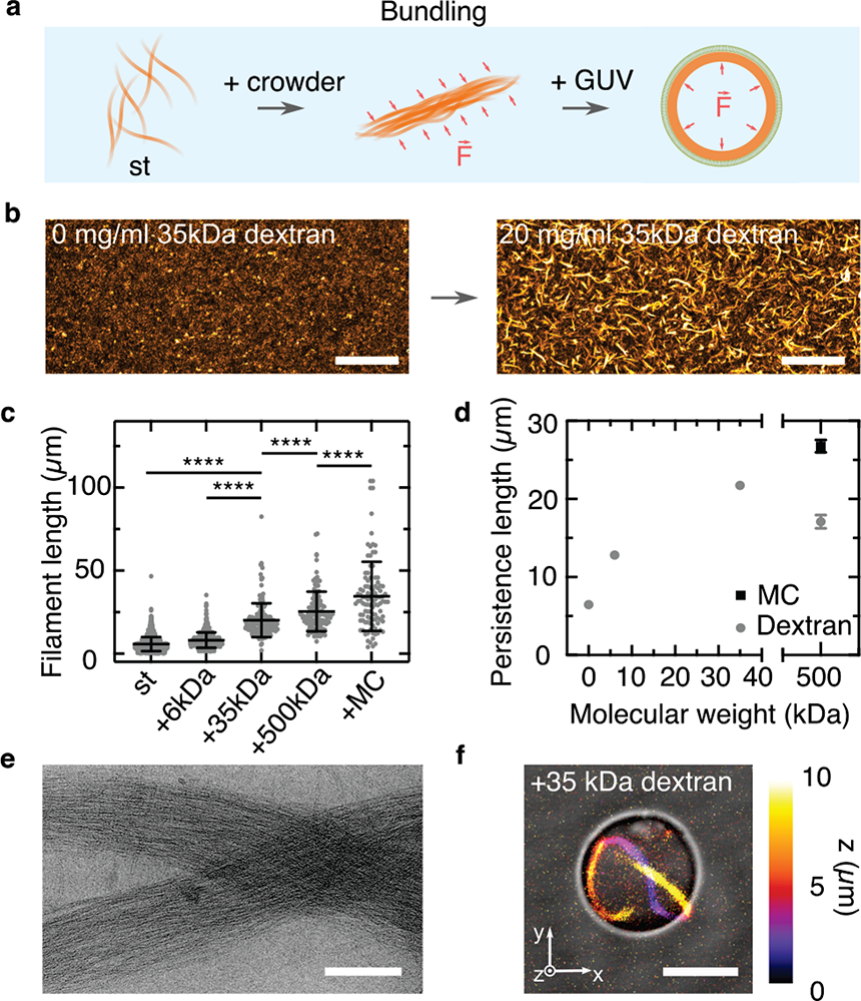
DNA filament bundling leads to the formation of ring-like structures within GUVs. (a) Schematic representation of the bundling of DNA filaments caused by the addition of a molecular crowder and the subsequent formation of a cortex-like network inside GUVs. (b) Confocal *z*-projection of st DNA filaments (orange, labeled with Cy3, λ_ex_ = 561 nm) in the absence and presence of 20 mg/mL 35 kDa dextran. Scale bar: 50 μm. (c) Length distribution of DNA filaments in the absence of bundling agents (st, *n* = 1896) and st DNA filaments in the presence of 20 mg/mL 6 kDa dextran (*n* = 510), 35 kDa dextran (*n* = 180), 500 kDa dextran (*n* = 129), and 500 kDa methylcellulose (MC, *n* = 104). All conditions are significantly different in length (*p* ≤ 0.001). (d) Persistence length of DNA filaments over molecular weight of the crowder (*n* = 11–15, mean ± SD). (e) Cryo-electron micrograph of bundled st filaments in the presence of 20 mg/mL dextran (MW = 35 kDa). Scale bar: 200 nm. (f) Overlay of color-coded confocal *z*-projection and bright-field image of 50 nM st filaments in the presence of 20 mg/mL 35 kDa dextran inside a GUV. Scale bar: 5 μm.

Inspired by cytoskeletal cortex formation which modulates cell morphology and stiffness, we engineer DNA filament cortices on the inner GUV membrane to modulate the stiffness and the morphology of the GUVs. Cortex formation can, in principle, be achieved by physical or chemical means, namely, by increasing the filament’s persistence length above the diameter of the compartment or by introducing chemical interactions with the lipid membrane. For example, actin filaments are bundled during cell division, which increases their persistence length and thus supports the formation of actin rings.^30^ We achieve bundling of DNA filaments based on the depletion effect by addition of molecular crowders (20 mg/mL, Figure 2a). We find that the addition of macromolecular dextran (Figure S14), methylcellulose (MC, Figure S15), as well as polyethylene glycol (Movie S2) completely changes the appearance of the DNA filaments. They bundle into tens of micrometers long rigid filaments, whereby the length depends on the chemical nature of the crowder as well as on its molecular weight (Figure 2b). Filaments bundled with methylcellulose (MC) are significantly longer than dextran-bundeled filaments at the same molecular weight of the crowder (500 kDa), but both bundling agents cause a significant increase in filament length compared to the bare st filaments (6.8 ± 4.3 vs 34.6 ± 20.8 μm in the presence of MC, Figure 2c). Importantly, we can tune the bundle length using dextran of different molecular weights (6, 35, or 500 kDa), yielding filaments with lengths of 8.1 ± 4.6, 20.2 ± 10.2, and 25.4 ± 12.0 μm, respectively. The bundling process influences not only the length but also the persistence of the DNA filaments: a larger molecular weight of the crowder generally leads to increased filament length and increased persistence length with values of up to 26.8 ± 0.8 μm (Figure 2d). The persistence length of the DNA filaments was calculated by tracing the filaments coordinates and calculating the average tangent correlation (see Experimental Section and Figure S16). This indicates that the filaments indeed form bundles, which we verify with cryo- and transmission electron microscopy. The DNA filament bundles comprise hundreds of individual DNA filaments, which are aligned with a high degree of order. The bundles have an average diameter of 418 ± 144 nm in the presence of 35 kDa dextran (Figure 2e and Figures S17 and S18). Note that even though the persistence length of encapsulated bundled DNA filaments (21.7 μm) is very similar to the one of actin filaments (17.7 μm),^31^ their polymerization rate is 1 order of magnitude lower (6 × 10^5^/M/s^32^ vs 75 × 10^5^/M/s^33^). This makes it less likely to observe protrusions from within the GUV as reported for actin filaments.^1^

Next, we reconstitute the bundled DNA filaments inside GUVs. We choose dextran as a crowding agent, as formation of GUVs in the presence of MC was not successful. In particular, the fusion process of the SUVs at the droplet periphery was inhibited, likely due to the higher viscosity of MC (Figure S19).^34^ We choose dextran with a molecular weight of 35 kDa because the resulting persistence length of 21.7 ± 0.6 μm is maximal and matches the GUV diameter. After DNA filaments are encapsulated in the presence of dextran, the large persistence length and the depletion effect cause the DNA filament bundles to localize and condense at the GUV periphery (Figure S20 and Movie S3). Moreover, for GUVs with a diameter below 15 μm, i.e., smaller than the persistence length of the DNA bundle (20.2 ± 10.2 μm), we achieve the reproducible formation of ring-like structures inside the GUVs around their circumference (Figure 2f and Figures S20 and S21). By employing st-azo tiles for DNA bundle formation, we can also induce the bundle disassembly within GUVs using UV illumination, although only after longer illumination times (Figure S22).

We have thus realized the formation of DNA filament bundles and reconstituted ring formation based on these entirely synthetic building blocks inside GUVs.

### Formation of DNA Cortex-Like Networks within GUVs

In cells, ring formation requires bundling of filaments, whereas membrane deformation relies on a link between the actin filaments and the cell’s periphery to establish cell shape or to form protrusions during cell migration.^35,36^

**Figure 3:**
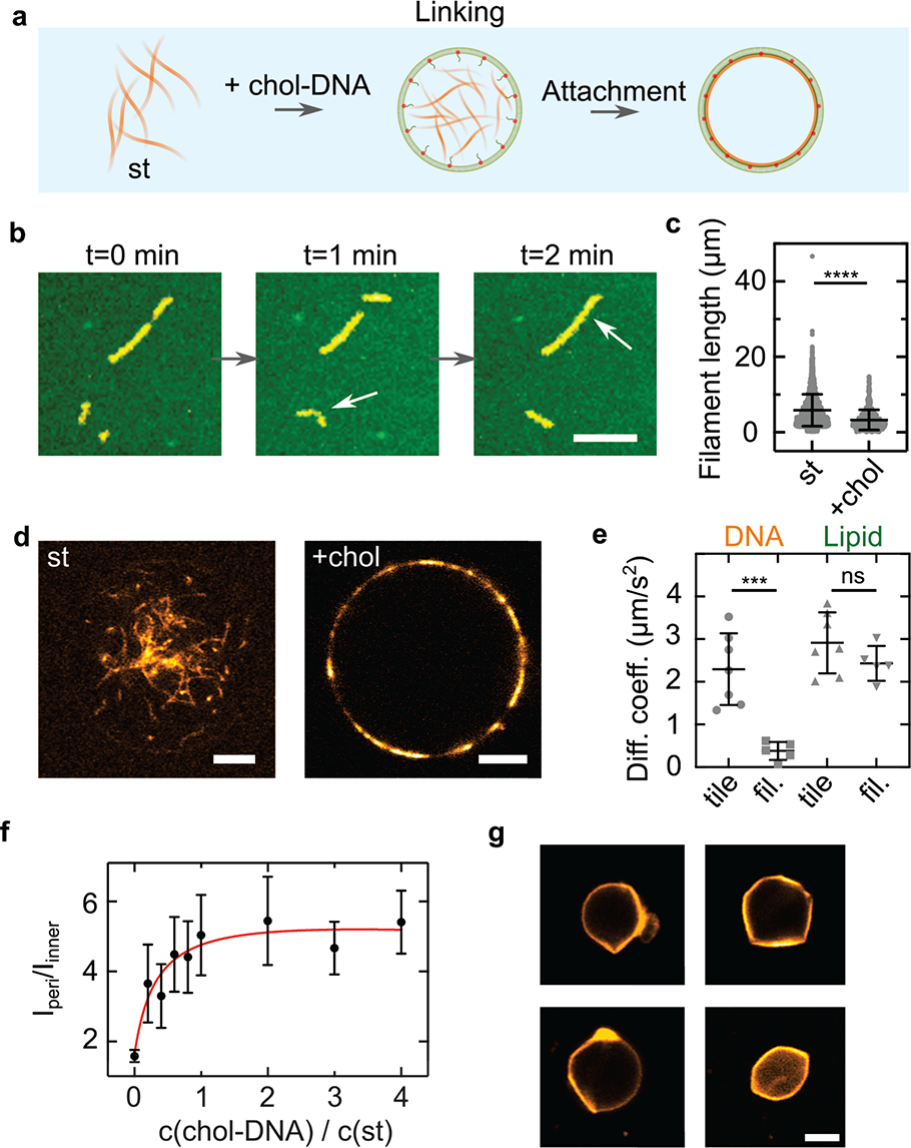
Deformation of GUVs from within by a membrane-linked DNA cortex-like network. (a) Schematic illustration of the linkage of DNA filaments to the GUV membrane with cholesterol-tagged DNA. (b) Confocal images of cholesterol-linked DNA filaments (st-chol, Cy3, λ_ex_ = 561 nm) on a supported lipid bilayer (SLB, green, Atto488-DOPE, λ_ex_ = 488 nm). The st-chol filaments diffuse and grow on the SLB. Scale bar: 10 μm. (c) Length distribution of st DNA filaments on glass (*n* = 1896) and st-chol-DNA filaments on SLBs (*n* = 429). The st-chol filaments are significantly shorter (*p* ≤ 0.001, mean ± SD). (d) Confocal images of 500 nM st (left) and st-chol filaments inside GUVs. For st-chol-containing GUVs, SUVs were incubated with 2 μM chol-link DNA for 2 min. Scale bar: 5 μm. (e) Diffusion coefficients of DNA filaments on SLBs determined by FRAP. Disassembled st-chol filaments (tile) exhibit 6-fold increased diffusion speeds compared to polymerized st-chol filaments (fil., *p* = 0.0007). The diffusion of the lipids of the SLB is not influenced by the polymerization state of the DNA filaments (*n* = 5–7, mean ± SD). (f) Fluorescence ratio *I*_peri_/*I*_inner_ of 500 nM st-chol filaments inside GUVs at varying chol-DNA to st ratios (*n* = 10–18 analyzed GUVs). (g) Confocal images of deformed GUVs containing 1 μM st-chol filaments at an osmolarity ratio of *c*_out_/*c*_in_ = 600 mOsm/300 mOsm = 2. Scale bar: 5 μm. (h) Circularity of deflated (*c*_out_/*c*_in_ = 600 mOsm/300 mOsm = 2) and undeflated (*c*_out_/*c*_in_ = 300 mOsm/300 mOsm = 1) GUVs containing 1 μM st-chol filaments (*n* = 4 and *n* = 7, respectively, mean ± SD, *p* = 0.008).

In analogy, to establish GUV shape with a DNA-based cortex-like network, we link the DNA cytoskeleton to the membrane with cholesterol-tagged DNA (chol-DNA). For this purpose, one of the strands of the st was extended with a single-stranded DNA overhang. A sequence complementary cholesterol-tagged DNA is added to the SUVs that fused at the droplet interface during GUV formation. In this way, the chol-DNA localizes at the inner bilayer leaflet of the GUV and serves as an attachment point for the filaments (Figure 3a). To first of all verify that the membrane-bound DNA filaments are intact on the membrane, we form a supported lipid bilayer (SLB) and functionalize it with the chol-DNA. With confocal microscopy, we verify the successful binding of st-chol-DNA filaments to the SLB. We find that DNA filaments are diffusive and even undergo membrane-assisted growth and occasionally breakage (Figure 3b and Movie S4). On average, st-chol-DNA filaments on an SLB are smaller than bare st DNA filaments (*l* = 3.3 ± 2.7 μm vs *l* = 6.8 ± 4.3 μm), likely due to steric hindrance or the additional electrostatic and diffusive forces acting on the filaments once they are bound to the membrane (Figure 3c). Similar to the case of DNA bundling, we observe the formation of a DNA filament cortex-like structure underneath the inner GUV membrane when the DNA filaments are linked with chol-DNA (Figure 3d and Movie S5). Interestingly, we also observe a significantly higher yield of GUVs (≈8000 GUVs/μL for st-chol and ≈1500 GUVs/μL for st) with our droplet-stabilized GUV method,^23^ indicating a mechanical stabilization of the GUVs (Figure S23). As shown in Figure 3e, fluorescence recovery after photobleaching (FRAP) confirms the presence of intact DNA filaments on the GUV membrane, which yield 6-fold lower diffusion coefficients of *D*_filament_ = 0.38 ± 0.21 μm^2^ s^–1^ compared to those of unpolymerized cholesterol-anchored DNA tiles (*D*_tile_ = 2.3 ± 0.8 μm^2^ s^–1^). Additionally, we confirm that lipid diffusion is only weakly affected by the presence of the DNA cortex-like network (*D*_lipid,tile_ = 2.9 ± 0.7 μm^2^ s^–1^ vs *D*_lipid,filament_ = 2.4 ± 0.4 μm^2^ s^–1^, *p* = 0.17). By changing the amount of cholesterol-tagged DNA at the GUV periphery, we tune the degree of DNA cortex-like network formation (Figure 3f and Figure S24). The degree of DNA cortex-like structure formation can be quantified by the ratio of the DNA filament intensity on the membrane, *I*_peri_, over the filament intensity in the GUV lumen, *I*_in_.^37^ Cortex-like network formation is enhanced at higher concentrations of chol-DNA and saturates when the chol-DNA is supplied at a ratio of 1:1 compared to the DNA tiles.

Ultimately, a membrane-linked DNA cortex-like network should be capable of establishing GUV shape.^7^ For this purpose, we use 2-fold higher amounts of DNA filaments (1 μM) and deflate the GUVs to an omsolarity ratio of *c*_out_/*c*_in_ = 2. Deflation provides sufficient excess membrane area to allow for GUV deformation. Figure 3g depicts representative examples of the successful deformation of GUVs from within. The internal DNA cortex-like network establishes the GUV shape. The GUVs often exhibit straight segments, which likely correspond to straight DNA filaments aligning at their periphery. To quantify the degree of deformation, we analyze the GUV circularity and find that deflated GUVs are significantly less spherical than undeflated GUVs with circularities of 0.955 ± 0.013 and 0.995 ± 0.001, respectively (Figure 3h). GUVs also remain more static in their deformed shape over time (Movie S6), again confirming their mechanical stabilization. In contrast, deflated GUVs that contain st DNA filaments without the chol-DNA handles exhibit apparent membrane fluctuations (Movie S7 and Figure S25).

Previously, GUVs were deformed externally with multilayer DNA origami structures only.^37−39^ However, it remained unclear whether DNA filaments, or DNA tile structures, in general, are sufficiently rigid to deform membranes. Furthermore, it was unclear whether deformation can be achieved from within the GUV, where the confined volume limits the amount of DNA that is available for membrane attachment. Hence, in addition to ring formation, we have implemented another pivotal characteristic of cytoskeletal filaments inside synthetic cells, namely, their linkage to the inner membrane for mechanical support to stabilize nonspherical GUV shapes.

## Conclusion

In summary, we have engineered programmable, versatile, and functional cytoskeletons made from DNA inside GUVs as synthetic cell models. Despite recent progress in the assembly of GUVs and the reconstitution of natural cytoskeletal filaments, it can be difficult to purify or coencapsulate a multitude of necessary proteins or to engineer them for versatile types of functions. Here, we achieved a diverse set of functions based on nucleic acids as engineerable and inherently biocompatible molecular building blocks and reconstitute them inside GUVs. Furthermore, we showed that by adapting the DNA tile design DNA filaments with a variety of customized functions can be obtained. These include the reversible light-mediated filament assembly and disassembly, DNA bundles with precise persistence lengths to trigger the formation of ring-like structures, and the formation of DNA cortices that can deform GUVs from within. Notably, these are only a few examples of conceivable functions of DNA-based cytoskeletons due to the variety of possible DNA structures. In the future, it will be especially exciting to equip DNA filaments with molecular motors for intracellular cargo transport, force generation and contractility. Moreover, the encapsulation of DNA filaments into GUVs sets a milestone for the reconstitution of any of the other DNA-based components that have already been developed for synthetic cells.^40^ It should be straightforward to use the same droplet-stabilized GUV for all of these components, which have rarely been implemented in confinement. All in all, DNA nanotechnology proves to be a versatile tool to build various functional modules for synthetic cells. Their inherent compatibility and the here demonstrated possibility to reconstitute them inside GUVs raise the prospects for a synthetic cell that consists of merely de novo synthesized parts. It will be exiting to witness if fully de novo assembled synthetic cells may even be achieved before their counterparts consisting of biological building blocks.

## Experimental Section

### DNA Tile Design and Assembly

DNA filament sequences were adapted from Rothemund et al.^18^ The individual DNA oligomers (5 per tile) were mixed to a final concentration of 5 μM in 10 mM Tris (pH 8), 1 mM EDTA, 12 mM MgCl_2_, and 5 mM NaCl. A total of 67–200 μL of the solution was annealed using a thermocycler (Bio-Rad) by heating the solution to 90 °C and cooling it to 25 °C in steps of 0.5 °C for 4.5 h. The assembled DNA filaments were stored at 4 °C and used within a week after annealing. The DNA strands were purchased from either Integrated DNA Technologies or Biomers (purification: standard desalting for unmodified DNA oligomers, HPLC for DNA oligomers with modifications). For DNA tiles that do not assemble into nanotubes, we used one of the two tiles from the tt DNA filament design, which could thus not form nanotubes. All DNA sequences are listed in Tables S1–S3.

### Confocal Fluorescence Microscopy

A confocal laser scanning microscope LSM 880 or LSM 900 (Carl Zeiss AG) was used for confocal microscopy. The pinhole aperture was set to one Airy Unit, and the experiments were performed at room temperature. Images of DNA filaments in GUVs in Figure 1 and Movie S1 were acquired using the Airyscan mode. The images were acquired using a 20× (Plan-Apochromat 20×/0.8 air M27, Carl Zeiss AG) or 63× objective (Plan-Apochromat 63×/1.4 oil DIC M27). Images were analyzed and processed with ImageJ (NIH, brightness and contrast adjusted).

### Analysis of DNA Filament Length

Annealed DNA filaments were diluted in 1× phosphate-buffered saline (PBS, Thermo Fisher) and 10 mM MgCl_2_ to a final concentration of 5 nM. DNA filaments were imaged in an untreated observation chamber made from two glass coverslides. Filaments attached to the glass slide via electrostatic interactions due to the presence of divalent magnesium ions in the buffer. Most of the images were analyzed using the Ridge Detection plugin in ImageJ. The parameters were chosen depending on the contrast of the image in a range from 1.15 to 2 for σ, from 0 to 5 for the lower threshold, and from 26 to 28 for the upper threshold. Some long filaments were manually analyzed using the ImageJ plugin FilamentJ.

### Cryo-Electron Microscopy

Samples were prepared for cryo-EM by applying 5 μL of sample solution (1× PBS, 10 mM MgCl_2_, 1 μM DNA filaments) onto a glow-discharged 300 mesh Quantifoil holey carbon-coated R3.5/1 grid (Quantifoil Micro Tools GmbH, Großlöbichau). The grid was blotted for 3 s and plunge-frozen in liquid ethane using a Vitrobot Mark IV (FEI NanoPort, Eindhoven, The Netherlands) at 100% humidity and stored under liquid nitrogen. Cryo-EM specimen grids were imaged on a FEI Tecnai G2 T20 twin transmission electron microscope (FEI NanoPort, Eindhoven, The Netherlands) operated at 200 kV. Electron micrographs were recorded with an FEI Eagle 4k HS, 200 kV CCD camera with a total dose of ≈40 electrons/Å^2^. Images were acquired at 50000× nominal magnification.

### Transmission Electron Microscopy

For negative staining, 5 μL of DNA filament-containing solution (1× PBS, 10 mM MgCl_2_, 1 μM DNA filaments, 20 mg/mL dextran or 0.4 wt % MC) of 0.5% PFA was applied onto a glow-discharged 100 mesh copper grid with carbon-coated Formvar (Plano GmbH, Wetzlar, Germany) and removed after 2 min by gentle blotting from one side with filter paper. The grid was rinsed with three drops of water, blotted again, and treated with 10 μL of 0.5% (w/v) uranyl acetate solution for 20 s. After the staining solution was thoroughly removed by blotting with filter paper, the grid was air-dried and imaged on a FEI Tecnai G2 T20 twin transmission electron microscope (FEI NanoPort, Eindhoven, The Netherlands) operated at 200 kV. Electron micrographs were acquired with an FEI Eagle 4k HS, 200 kV CCD camera at 20000× nominal magnification.

### Preparation of Small Unilamellar Vesicles

Lipids were stored in chloroform at −20 °C and used without further purification. Small unilamellar vesicles (SUVs) were formed by mixing the chloroform-dissolved lipids (69% 1,2-dioleoyl-*sn*-glycero-3-phosphocholine (DOPC, Avanti Polar Lipids), 30% 1,2-dioleoyl-*sn*-glycero-3-phospho-(1′-*rac*-glycerol) (sodium salt, DOPG, Avanti Polar Lipids) and 1% Atto488-1,2-dioleoyl-*sn*-glycero-3-phosphoethanolamine (Atto488-DOPE, Atto TEC)) in a glass vial. The lipid solution was dried under a stream of nitrogen gas. To remove traces of solvent, the vial was kept under vacuum in a desiccator for at least 20 min. Lipids were resuspended in 10 mM Tris (pH 8) and 1 mM EDTA at a final lipid concentration of 2.5 mM. The solution was vortexed for 10 min to trigger vesicle formation. Subsequently, vesicles were extruded to form homogeneous SUVs with 11 passages through a polycarbonate filter with a pore size of 50 nm (Avanti Polar Lipids, Inc.). SUVs were stored at 4 °C for up to a week or used immediately for GUV formation.

### Preparation of Giant Unilamellar Vesicles

Giant unilamellar vesicles (GUVs) were formed using the droplet-stabilized GUV formation method.^23^ Briefly, 1.25 mM SUVs, DNA filaments made from 500 nm DNA tiles (if not stated otherwise), 10 mM MgCl_2_, and phosphate-buffered saline (PBS consisting of 137 mM NaCl, 2.7 mM KCl, 10 mM Na_2_HPO_4_, and 1.8 mM KH_2_PO_4_) were mixed together. The aqueous mix was layered on top of an oil-surfactant mix containing 1.4 wt % perfluoropolyether–polyethylene glycol (PFPE–PEG) fluorosurfactants (Ran Biotechnologies) and 10.5 mM PFPE–carboxylic acid (Krytox, MW 7000–7500 g/mol, DuPont) in a microtube (Eppendorf). The ratio between the aqueous and oil phase was 1:3, generally leading to volumes of 100/300 μL. Droplet-stabilized GUVs were generated by shaking the microtube vigorously by hand. The water-in-oil emulsion droplets were left at room temperature for 1–2 h. Within this incubation period, the SUVs fused at the droplet periphery to create a spherical supported lipid bilayer, termed droplet-stabilized GUV. Afterward, the oil phase was removed, and 100 μL of 1× PBS was added on top of the emulsion droplets. The droplet-stabilized GUV was destabilized by addition of 100 μL of perfluoro-1-octanol (PFO, Sigma-Aldrich) to release free-standing GUVs into the PBS. GUVs were stored for up to 2 days at 6 °C. GUVs were imaged in a custom-built observation chamber that was coated with 2 mg/mL bovine serum albumin (BSA, Sigma-Aldrich) for 15 min to prevent fusion of the GUVs with the glass coverslide. Additionally, DNA filaments in the outer aqueous solution due to an imperfect release were disassembled due to the absence of MgCl_2_ in the release buffer and the addition of 4 μM of an invader strand (Table S3) that leads to filament disassembly by a toehold-mediated strand displacement reaction.

### Light-Mediated Disassembly of DNA Filaments

Light-mediated disassembly was achieved by incorporating two azobenzene modifications at the sticky overhangs of the S4 strand (Table S3), positioned two bases before the 3′ and 5′ ends.^43,44^ This breaks up the sticky end sequence into two segments with 2 or 3 bases each, which renders them able to hybridize in the presence of *trans*-azobenzene and unstable with *cis*-azobenzene. GUVs with azobenzene-modified DNA filaments were formed as before. Disassembly was achieved by illumination with full power of a GUV with a 100 W mercury lamp (Zeiss) using a DAPI filter set (excitation: 365 nm/bandwidth: 10 and an emission window starting at 420 nm, Filter Set 02, Zeiss) for 15 s. Subsequently, filaments reassembled within 30 min before they were illuminated again with a mercury lamp (HBO 100). The confocal images of the DNA filaments were thresholded in ImageJ using Otsu’s method, and their overall porosity was analyzed. Note that the porosity indicates the degree of polymerization of the DNA filaments. The porosity values were corrected for bleaching by determining the slope of linear fits *x*_slope_ for the porosity of disassembled states. This leads to the corrected values: *p*_corr_ = *p* × (1 − *x*_slope_*t*).

### Bundling of DNA Filaments

Bundling of DNA filaments was induced by addition of molecular crowders like dextran (molecular weight: 6, 35, and 500 kDa, Carl Roth), polyethylene glycol (PEG, molecular weight 8 kDa, Carl Roth), or methylcellulose (molecular weight: 500 kDa, Carl Roth). The bundling agent was mixed with DNA filaments directly before GUV formation. For transmission electron microscopy and confocal imaging of DNA bundles in bulk, DNA filaments were incubated for 5 min with the respective bundling agent.

### Determination of the DNA Filament’s Persistence Length

For the persistence length analysis, DNA filaments with a length around the mean value of the filament length of each condition were considered. DNA filaments were traced, and filament coordinates were extracted using an automated tracing algorithm.^41^ The coordinates had a unit spacing of Δ*s* = 4 pixels = 0.62 μm. Subsequently, we calculated the average tangent correlation ⟨*t̂*(*x*) × *t̂*(*x* + Δ*x*)⟩ for each filament using a custom-written Python script. We then averaged the tangent correlation for each distance between the tangents Δ*x* from all considered filaments. The resulting values were used to fit a function of the form ⟨*t̂*(*x*) × *t̂*(*x* + Δ*x*)⟩ = *e*^–Δ*x*/2*P*^ from Δ*x* = 2 to Δ*x* = 8 to obtain the filament’s persistence length, *P*. The principle of this persistence length analysis is based on previous work with DNA filaments.^42^

### Linking of DNA Filaments to Supported Lipid Bilayers

SUVs were diluted in 1× PBS containing 10 mM MgCl_2_ to a final lipid concentration of 1 mM and flushed into an untreated observation chamber. The chamber was sealed, and the SUVs were left to fuse with the coverslide for 1 h. The chamber was then opened up and flushed twice with deionized water to remove remaining SUVs that did not fuse to form a supported lipid bilayer. Subsequently, 20 μL of 1× PBS and 2 μM of chol-DNA (chol-link, Table S3) were flushed in and incubated with the SLB for 10 min. Finally, 5 nM Cy3-labeled DNA filaments were added in 1× PBS and 10 mM MgCl_2_. Note that the single-stranded overhang on the S3 strand is complementary to the chol-link DNA. The chamber was sealed for confocal imaging.

### DNA Cortex-like Network Formation inside Giant Unilamellar Vesicles

For DNA cortex-like network formation, we designed a cholesterol-tagged complementary DNA (chol-link) that can hybdridize with a single-stranded DNA overhang on the S3 strand (Table S3). Before GUV formation, SUVs and 2 μM chol-link DNA were incubated for 2 min to bind the DNA filaments to the GUV periphery. The acquired images were analyzed by determining the peripheral intensity of the DNA filaments over the interior DNA filament intensity using a custom written ImageJ macro (available here: 10.5281/zenodo.4738934). GUV deformation was achieved by osmotic deflation of the GUVs such that the concentration of ions outside the GUV is increased by a factor of 2 (*c*/*c*_0_ = 2). After osmotic deflation, the DNA filaments inside the GUVs were reannealed in the thermocycler (BioRad).

### Fluorescence Recovery after Photobleaching

For fluorescence recovery after photobleaching (FRAP) experiments a circular region of interest (ROI) with a diameter of 10 μm at the top confocal plane of the GUV was chosen. By choosing the top of the GUV, we reduce the risk of measuring artifacts due to the interaction with the BSA-coated glass slide at the bottom. Three images were acquired before the ROI was illuminated for 100 iterations at the 100% laser power with 488 nm (for the Atto488-labeled lipids) or 561 nm (for Cy3-labeled DNA tiles and filaments). Afterward, the ROI was imaged for up to 60 s to track the recovery of the fluorescence. To quantify the diffusion coefficient, we used a custom-written Matlab (R2019a) script.^25^

### Statistical Analysis

All of the experimental data were reported as mean ± SD from *n* experiments, filaments, or GUVs. The respective value for *n* is stated in the corresponding figure captions. All experiments were repeated at least twice. To analyze the significance of the data, a Student’s *t* test with Welch’s correction was performed using Prism GraphPad (version 9.1.2), and *p* values correspond to ****: *p* ≤ 0.0001, ***: *p* ≤ 0.001, **: *p* ≤ 0.01, *: *p* ≤ 0.05, and ns: *p* > 0.05.

## Supporting Information Available

Figures S1–S25, with additional experimental results, and Tables S1–S3, containing the used DNA sequences (PDF)
Movie S1: Dynamics of st DNA filaments inside GUVs (MP4)
Movie S2: Bundling of st DNA filaments with polyethylene glycol as molecular crowder (MP4)
Movie S3: Formation of DNA cortex-like networks via bundling agents (MP4)
Movie S4: st-chol-DNA filaments diffuse on SLBs (MP4)
Movie S5: Formation of DNA cortex-like networks induced by cholesterol-tagged DNA-mediated linking (MP4)
Movie S6: GUVs are deformed by st-chol-DNA filaments (MP4)
Movie S7: Deflated GUV in the presence of st DNA filaments (MP4)
