# Supplementary material for: Bottom-Up Assembly of Synthetic Cells with a DNA Cytoskeleton
Source: ACS Nano. 2022 Apr 4;16(5):7233–41. doi: 10.1021/acsnano.1c10703 (PMC9134502; doi:10.1021/acsnano.1c10703)
Supplement: Supplementary file 1 — nn1c10703_si_001.pdf [file nn1c10703_si_001.pdf]

Supporting Information: Bottom-up Assembly of Synthetic Cells with a DNA Cytoskeleton

Kevin Jahnke[1,2], Vanessa Huth[1,2], Ulrike Mersdorf[3],

Na Liu[4,5], Kerstin Göpfrich[1,2*]

[1]Biophysical Engineering Group, Max Planck Institute for Medical Research,

Jahnstraße 29, D-69120 Heidelberg, Germany,

[2]Department of Physics and Astronomy, Heidelberg University,

D-69120 Heidelberg, Germany

[3]Department of Biomolecular Mechanisms, Max Planck Institute for Medical Research,

Jahnstraße 29, D-69120 Heidelberg, Germany,

[4] 2nd Physics Institute, University of Stuttgart,

Im Pfaffenwaldring 57, D-70569 Stuttgart, Germany

[5] Max Planck Institute for Solid State Research,

Heisenbergstraße 1, D-70569 Stuttgart, Germany

*

E-mail: kerstin.goepfrich@mr.mpg.de

Contents

Supporting Figures **5**

Supporting Figure 1: Cryo electron micrographs of st, tt and st-azo DNA filaments 5

Supporting Figure 2: Single-tile DNA filament diameter from cryo electron micrographs 6

Supporting Figure 3: Examples and overview of polymerized single-tile DNA filaments within GUVs 7

Supporting Figure 4: Single-tile DNA filaments inside water-in-oil droplets before the release of free-standing GUVs 8

Supporting Figure 5: DNA filaments with orthogonal two-tile design polymerize within GUVs 9

Supporting Figure 6: DNA filament assembly inside GUVs over time 10

Supporting Figure 7: tt DNA filament clustering due to the presence of Mg^{2+} . . 11

Supporting Figure 8: Schematic representation of the st overhang modified with azobenzene (st-azo) 12

Supporting Figure 9: Azobenzene-modified DNA filaments disassemble within GUVs after UV illumination 13

Supporting Figure 10: UV illumination does not affect unmodified DNA filaments inside GUVs 14

Supporting Figure 11: Unmodified st DNA filaments do not disassemble during UV illumination. 15

Supporting Figure 12: Analysis of the reversible assembly of azobenzene-modified DNA filaments within GUVs 16

Supporting Figure 13: Azobenzene-modified DNA filaments assemble reversibly within GUVs 17

Supporting Figure 14: Dextran induces bundling of st DNA filaments in bulk and in GUVs 18

Supporting Figure 15: Methylcellulose induces bundling of st DNA filaments in bulk 19
Supporting Figure 16: Persistence length analysis 20
Supporting Figure 17: Cryo electron micrographs of st DNA bundles formed by addition of dextran 21
Supporting Figure 18: TEM images of st DNA bundles formed by addition of methylcellulose 22
Supporting Figure 19: st DNA bundles formed by addition of methylcellulose cannot be reconstituted into GUVs 23
Supporting Figure 20: Confocal overlay of bundled st DNA filaments form ring-like structures inside GUVs 24
Supporting Figure 21: Bundled st DNA filaments form ring-like structures inside GUVs 25
Supporting Figure 22: Disassembly of bundled st-azo DNA filaments inside GUVs 26
Supporting Figure 23: GUV formation efficiency increases in presence of cholesterol-tagged DNA filaments 27
Supporting Figure 24: DNA filaments are recruited to the inner membrane of GUVs in the presence of cholesterol-tagged DNA 28
Supporting Figure 25: Membrane-bound DNA filaments suppress membrane fluctuations 29

Supporting Tables **30**

Supporting Table 1: DNA sequences for st DNA filaments 30
Supporting Table 2: DNA sequences for two-tile DNA filaments 30
Supporting Table 3: DNA sequences for modified DNA filaments 31

Supporting Movies **32**

Supporting Movie 1: Dynamics of st DNA filaments inside GUVs 32

Supporting Movie 2: Bundling of st DNA filaments with polyethylene glycol as molecular crowder 32
Supporting Movie 3: Formation of DNA cortex-like networks *via* bundling agents 32
Supporting Movie 4: st-chol DNA filaments diffuse on SLBs 32
Supporting Movie 5: Formation of DNA cortex-like networks induced by cholesterol-tagged DNA-mediated linking 33
Supporting Movie 6: GUVs are deformed by st-chol DNA filaments 33
Supporting Movie 7: Deflated GUV in presence of st DNA filaments 33

References **34**

Supporting Figures

Supporting Figure 1: Cryo electron micrographs of st, tt and st-azo DNA filaments

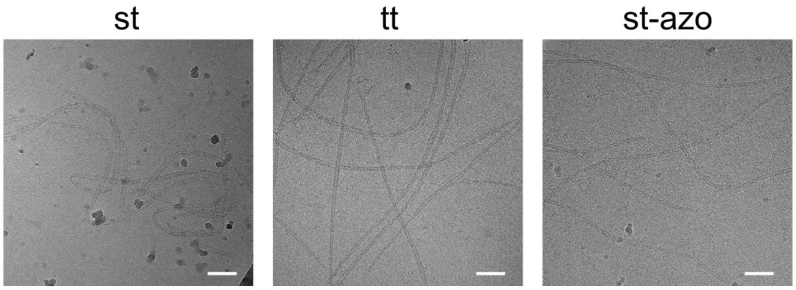

Figure 1: Cryo electron micrographs of st, tt and st-azo DNA filaments verifying the correct assembly of the respective DNA tiles into micrometer long filaments. Scale bars: 100 nm.

Supporting Figure 2: Single-tile DNA filament diameter from cryo electron micrographs

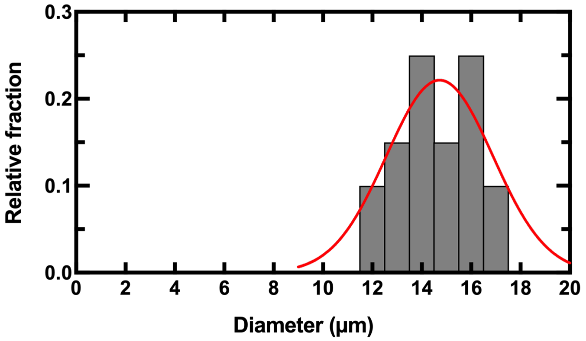

Figure 2: Distribution of single-tile (st) DNA filament diameters as determined from cryo electron micrographs (n = 20). Filament diameters were measured with the line profile tool in ImageJ. A Gaussian fit reveals a diameter of 14.5 ± 1.8 nm, consistent with the assembly of filaments consisting of 12-14 DNA duplexes.

Supporting Figure 3: Examples and overview of polymerized single-tile DNA filaments within GUVs

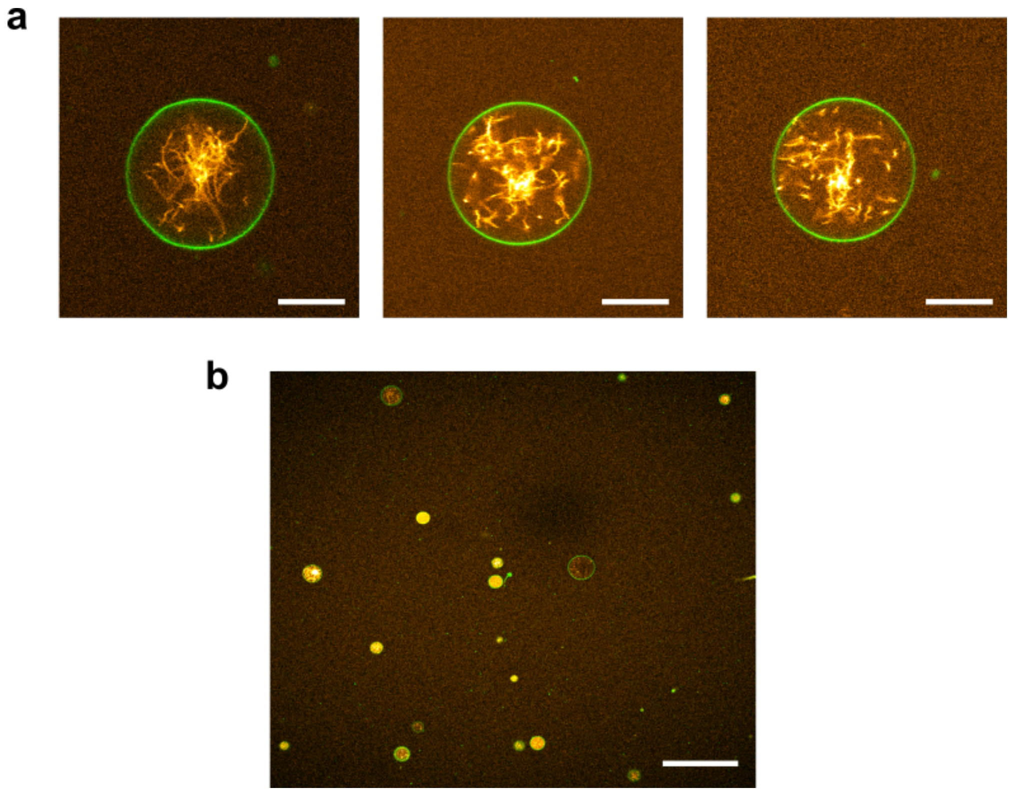

Figure 3: **a** Examples of polymerized single-tile (st) DNA filaments with within GUVs. Confocal fluorescence images of GUVs (green, λ_{ex} =488 nm) containing 500 nM st DNA filaments after GUV formation (orange, λ_{ex} =561 nm, see Supporting Table 1). Scale bars: 10 µm. **b** Overview confocal image of successful encapsulation of st DNA filaments into GUVs at high yield. GUVs (green, λ_{ex} =488 nm) contain 500 nM st DNA filaments (orange, λ_{ex} =561 nm). Scale bar: 100 µm.

Supporting Figure 4: Single-tile DNA filaments inside water-in-oil droplets before the release of free-standing GUVs

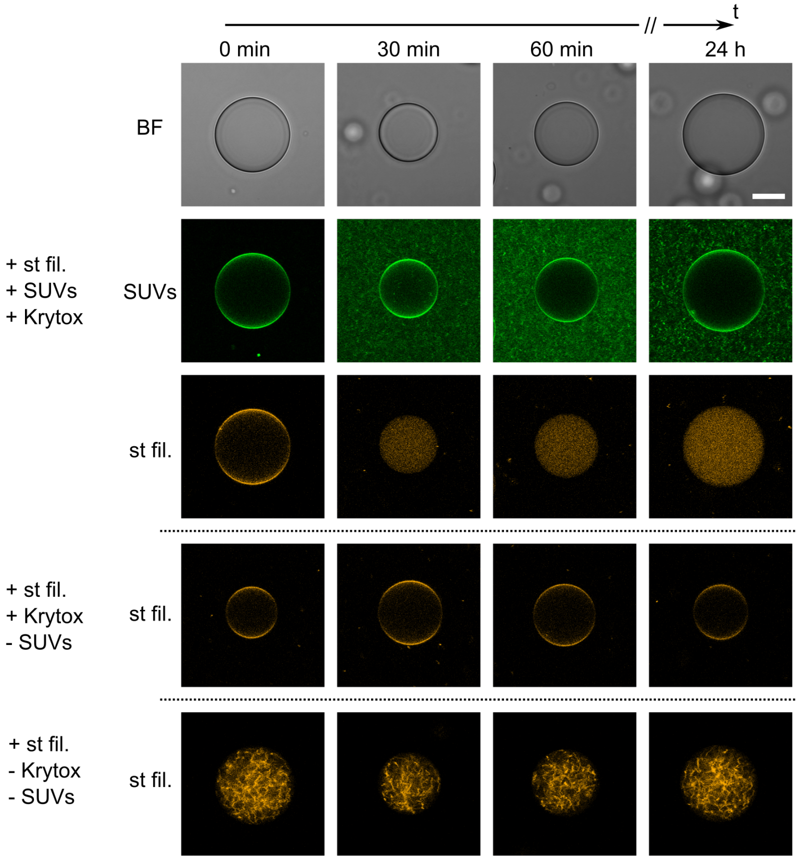

Figure 4: Confocal images of 500 nM st DNA filaments (orange, labelled with Cy3, λ_{ex} = 561 nm) within surfactant-stabilized water-in-oil droplets over time in presence of 1.25 mM lipids in the form of SUVs (green, 69% DOPC, 30% DOPG, 1% Atto488-DOPE, λ_{ex} = 488 nm) and 10.5 mM Krytox (top three rows), 10.5 mM Krytox (4th row) or in the absence of Krytox (bottom row). After the formation of a supported lipid bilayer at the droplet periphery, access SUVs are dragged out of the aqueous phase inside the droplet into the oil phase.[1] st DNA filaments are disassembled and initially localized at the droplet periphery but homogeneously distributed inside the droplet lumen after 30 min i.e. once exccess SUVs have been dragged out of the droplet-stabilized GUV. This suggests that st DNA tiles interact with the negatively charged Krytox at the droplet periphery. They are then displaced from the droplet periphery once the SUVs fused and created a droplet-stabilized GUV. This is also confirmed by the fact that the st DNA tiles remain at the droplet periphery in the absence of SUVs (4th row). The fact that the DNA filaments do not reassemble suggests that the presence of Krytox in the oil phase can lead to significant changes of the chemical environment inside water-in-oil droplets by possibly reducing the pH (Krytox has a carboxylic acid head group) or extracting ions by micellular exclusion. Scale bar: 10 µm.

Supporting Figure 5: DNA filaments with orthogonal two-tile design polymerize within GUVs

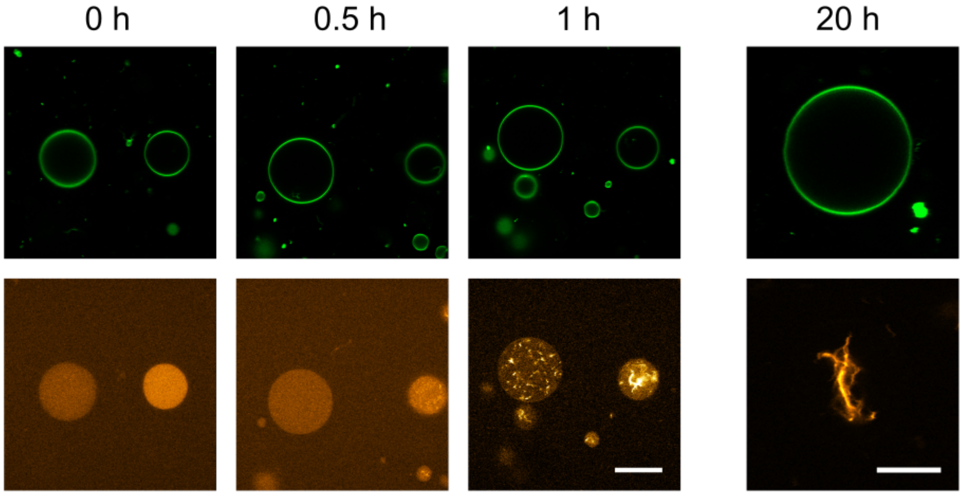

Figure 5: DNA filaments with orthogonal two-tile (tt) design polymerize within GUVs. Confocal fluorescence images of GUVs (λ_{ex} =488 nm, green) containing 250 nM RE and SE tiles (λ_{ex} =561 nm, orange, see Supporting Table 2). DNA filaments assemble within 20 h after GUV formation. Scale bars: 20 µm and 10 µm, respectively.

Supporting Figure 6: DNA filament assembly inside GUVs over time

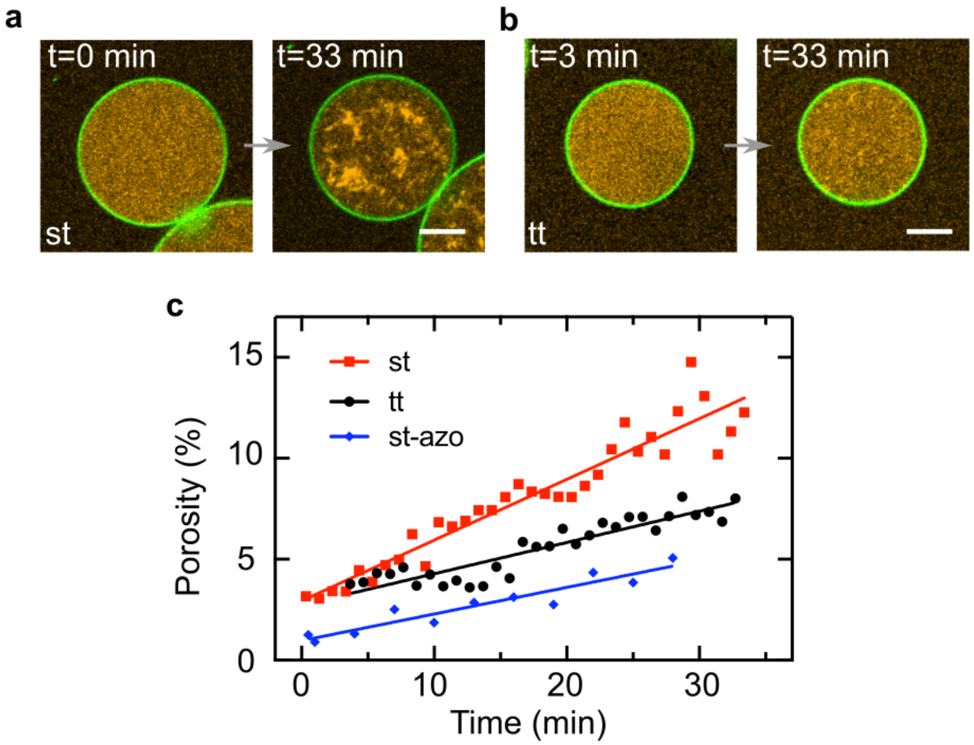

Figure 6: DNA filament assembly inside GUVs over time for st, tt and st-azo DNA filaments. **a** st DNA filaments (orange, λ_{ex} =561 nm) assemble within 30 min inside GUVs (green, λ_{ex} =488 nm) after the GUV formation process. Scale bar: 10 µm. Note that they are in a disassembled state prior to the GUV release from the water-in-oil droplets due to the presence of Krytox. **b** tt DNA filaments (orange, λ_{ex} =561 nm) do not fully assemble within 30 min inside GUVs (green, λ_{ex} =488 nm). Their assembly takes about 2-3 h. Scale bar: 10 µm. **c** Porosity over time for the assembly processes of st (red), tt (black) and st-azo (blue) DNA filaments inside GUVs. The data was fitted with a linear fit revealing slopes of $0.30 \pm 0.02\,\%\,\text{min}^{-1}$ (st), $0.15 \pm 0.01\,\%\,\text{min}^{-1}$ (tt) and $0.13 \pm 0.01\,\%\,\text{min}^{-1}$ (st-azo). This indicates an at least two-fold faster assembly for st DNA filaments compared to tt and st-azo DNA filaments due to the necessity of two tiles or azobenzene cis-trans isomerization for filament assembly.

Supporting Figure 7: tt DNA filament clustering due to the presence of Mg^{2+}

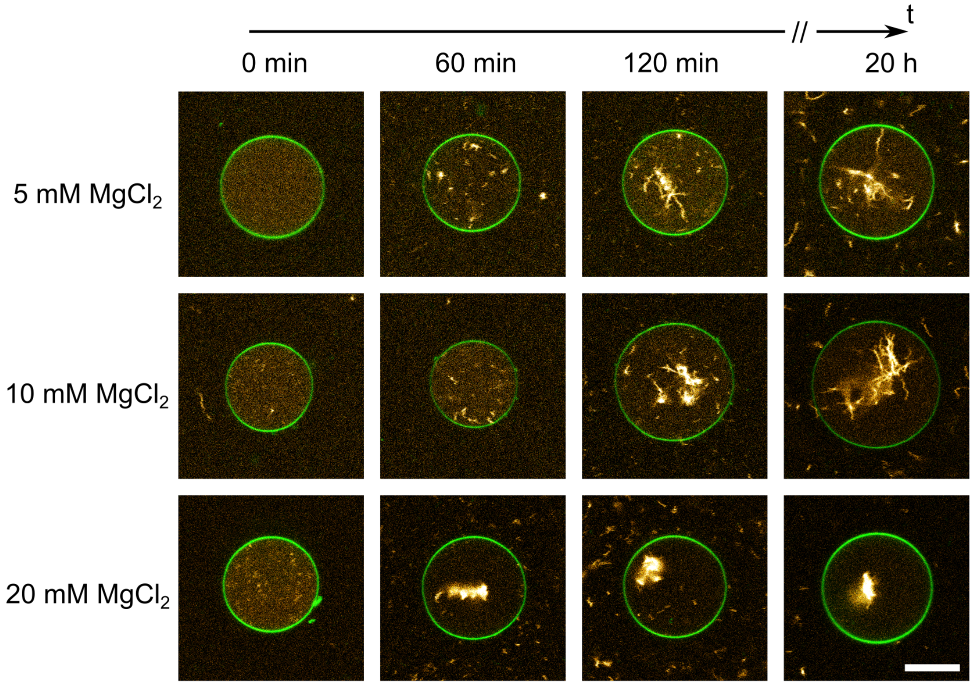

Figure 7: Confocal overlay images of 500 nM tt DNA filaments (orange, labelled with Cy3, $\lambda_{ex} = 561$ nm) encapsulated into GUVs (green, 69% DOPC, 30% DOPG, 1% Atto488-DOPE, $\lambda_{ex} = 488$ nm) in presence of 5 mM, 10 mM or 20 mM $MgCl_2$ at different time points. tt DNA filaments have slower polymerization kinetics compared to st DNA filaments. DNA filaments cluster over time with increasing Mg^{2+} concentrations. At 20 mM Mg^{2+}, the filaments form a clump within the droplet lumen. Note that we used 10 mM Mg^{2+} for almost all experiments in the manuscript due to the enhanced GUV formation compared to 5 mM Mg^{2+} (SUVs require Mg^{2+} to fuse to the droplet periphery ([1]). Scale bar: 10 µm.

Supporting Figure 8: Schematic representation of the st overhang modified with azobenzene (st-azo)

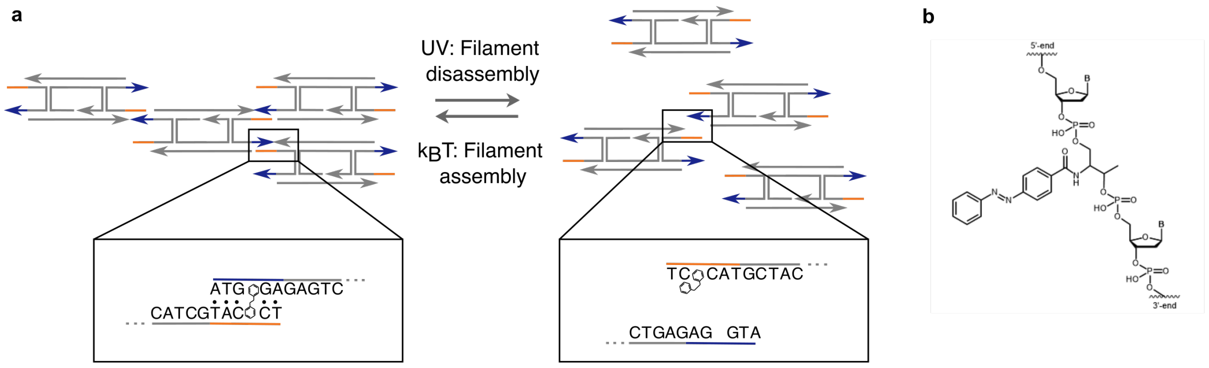

Figure 8: Schematic representation of the st overhang modified with azobenzene (st-azo) for reversible cytoskeleton assembly with UV light. **a** DNA filaments disassemble due to trans-cis isomerization induced *via* UV illumination and reassemble due to thermal cis-trans isomerization. **b** Chemical structure of the conjugation of azobenzene in between two bases at the DNA tile overhang.

Supporting Figure 9: Azobenzene-modified DNA filaments disassemble within GUVs after UV illumination

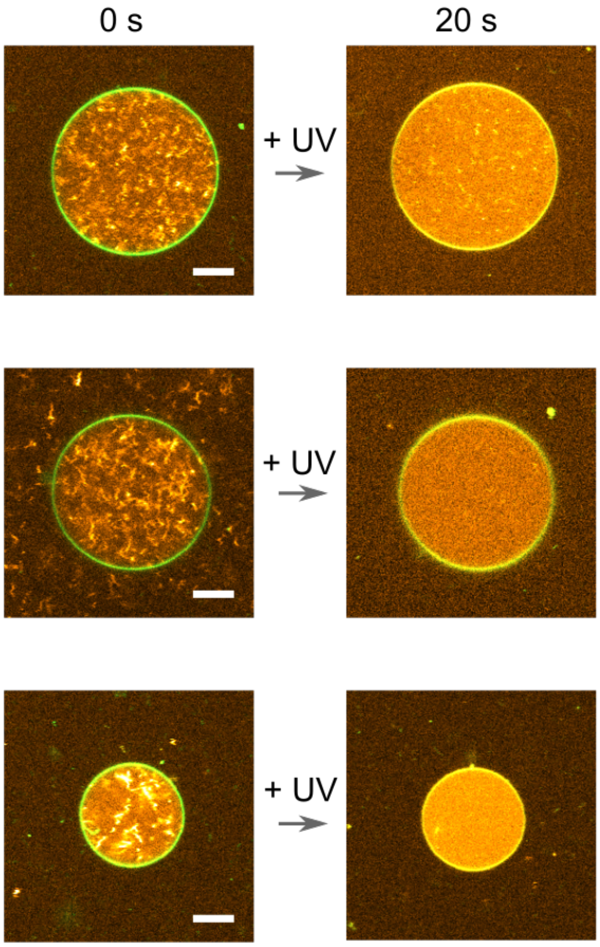

Figure 9: Azobenzene-modified DNA filaments (st-azo) disassemble within GUVs after 20 s of illumination with the UV laser of a confocal microscope. Confocal fluorescence images of GUVs (green, λ_{ex} =488 nm) containing 500 nM st-azo DNA filaments (orange, λ_{ex} =561 nm, for DNA sequences see Supporting Table 3). Confocal images are taken directly before (left column) and directly after the 20 s of UV illumination. Scale bar: 10 µm.

Supporting Figure 10: UV illumination does not affect unmodified DNA filaments inside GUVs

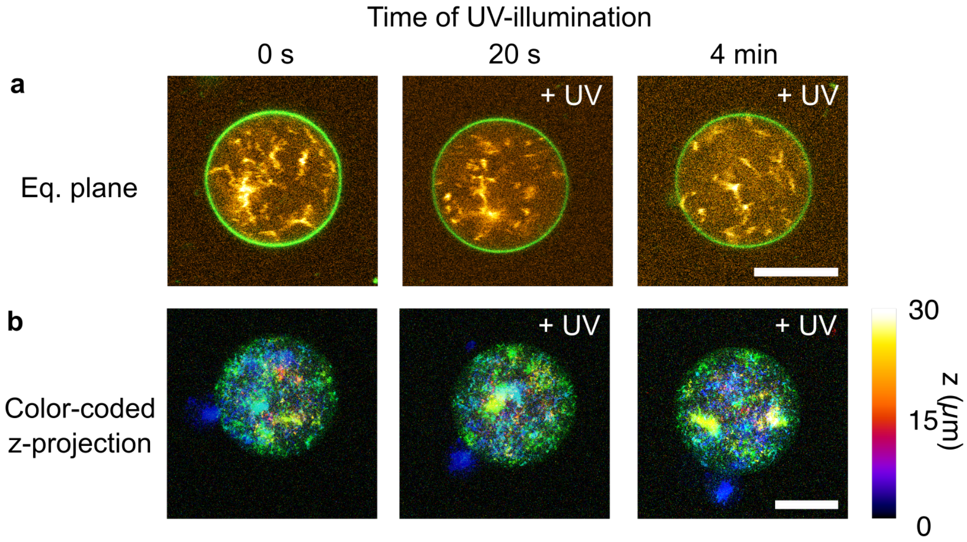

Figure 10: st filament disassembly upon UV-illumination is not observed in the absence of the azobenzene modification. Hence, UV illumination does not destroy st DNA filaments inside GUVs. Confocal images at different time points during UV-illumination show GUVs (green, λ_{ex} =488 nm) in the equatorial plane (**a**) or as color-coded z-projection (**b**). GUVs contain 500 nM unmodified st DNA filaments (orange, λ_{ex} =561 nm) during illumination with UV light for up to 4 min. Scale bar: 10 µm. While some bleaching is observed, the filaments do not disassemble without the azobenzene modification.

Supporting Figure 11: Unmodified st DNA filaments do not disassemble during UV illumination.

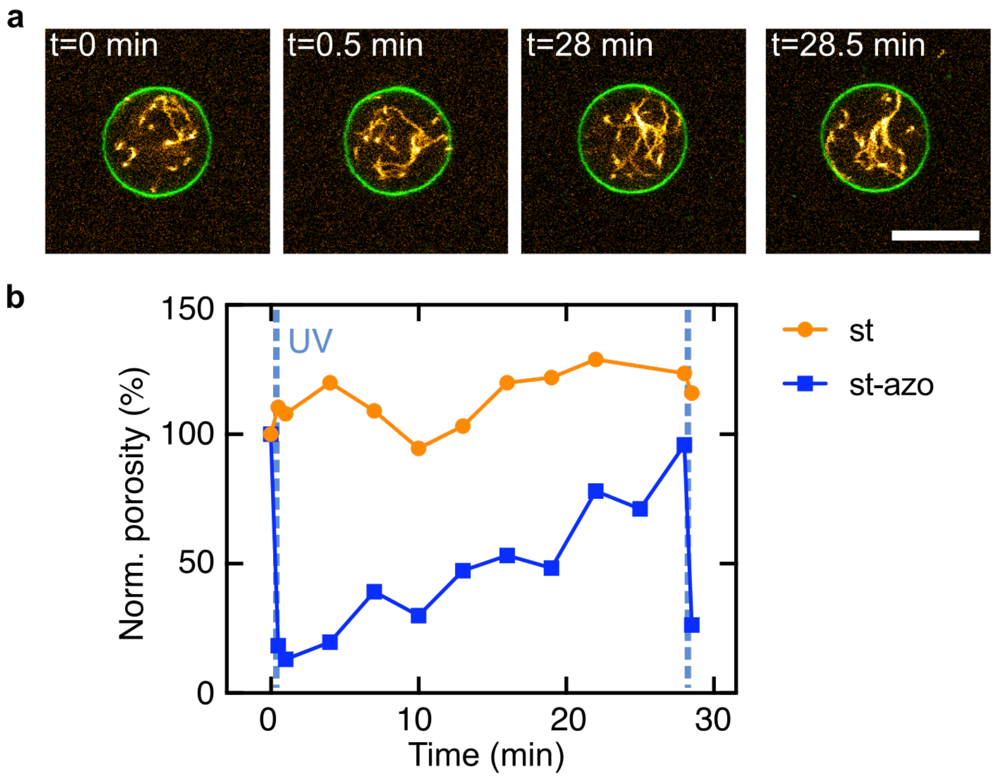

Figure 11: Unmodified st DNA filaments do not disassemble during UV illumination. **a** Confocal images of 500 nM st DNA filaments (orange, labelled with Cy3, $\lambda_{ex} = 561$ nm) encapsulated within GUVs (green, 69% DOPC, 30% DOPG, 1% Atto488-DOPE, $\lambda_{ex} = 488$ nm) before and after UV illumination. The GUV was illuminated with UV light for 20 s at 0 min and 28 min. Scale bar: 10 µm. **b** Normalized porosity over time of st and st-azo DNA filaments within GUVs. We do not detect any significant decrease in the porosity, i.e. filament disassembly, of st DNA filaments over time.

Supporting Figure 12: Analysis of the reversible assembly of azobenzene-modified DNA filaments within GUVs

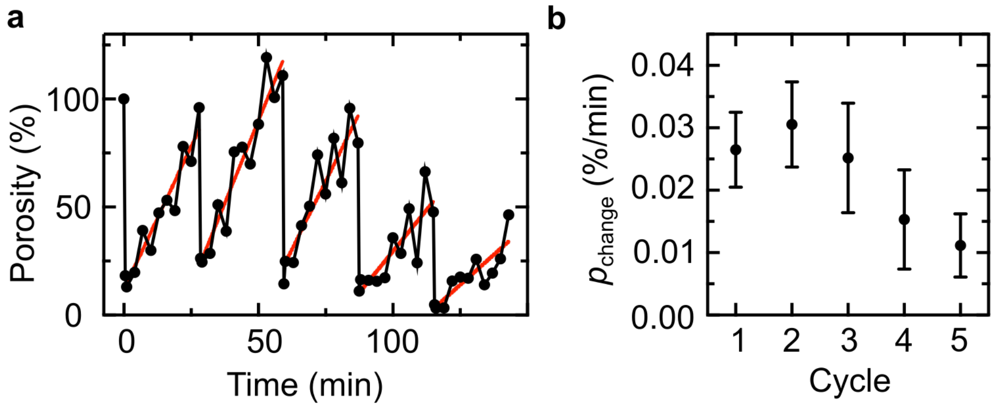

Figure 12: Analysis of the reversible assembly of azobenzene-modified DNA filaments (st-azo) within GUVs. **a** Porosity of DNA filament fluorescence, corresponding to the degree of filament polymerization, over time inside a GUV. Each reassembly process after UV illumination for 15 s was fitted (red lines) with a linear fit of the form $y = p_{\mathrm{change}}x + b$. **b** Average slope of the linear fit p_{change} during each disassembly-reassembly cycle reveals the reversible assembly of DNA filaments with no fatigue for the first three cycles with an average porosity change of $p_{\mathrm{change}} = 0.027 \pm 0.003\,\%\,\mathrm{min}^{-1}$. The decrease of the porosity change in subsequent cycles can likely be attributed to a combination of photobleaching, UV damage and trapped states of the isomerization process.

Supporting Figure 13: Azobenzene-modified DNA filaments assemble reversibly within GUVs

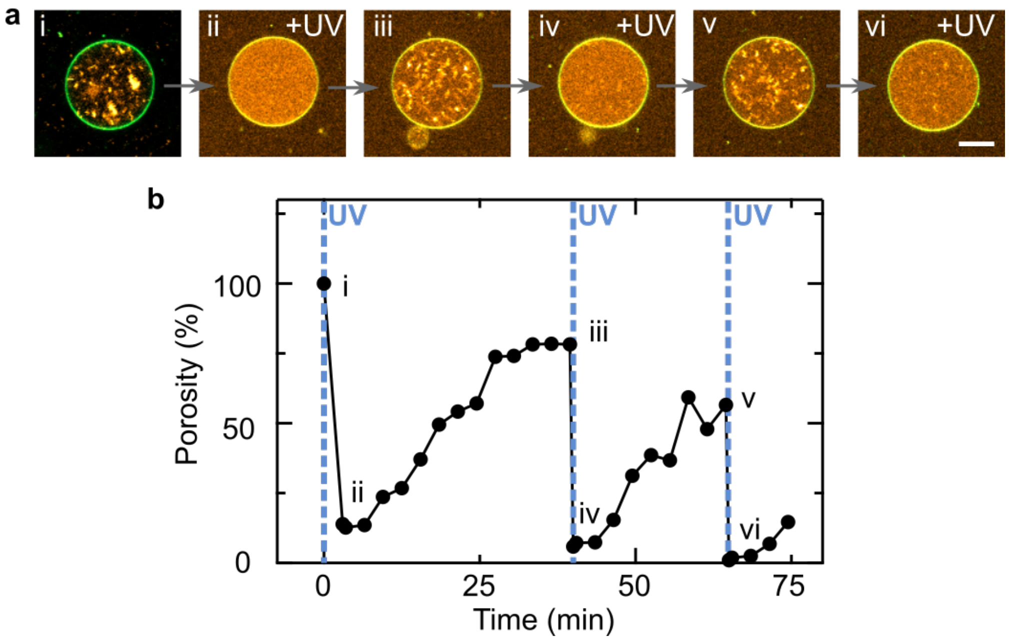

Figure 13: Azobenzene-modified (st-azo) DNA filaments assemble reversibly within GUVs. **a** Confocal fluorescence images of a GUV (green, λ_{ex} =488 nm) containing 500 nM st-azo DNA filaments (orange, λ_{ex} =561 nm, for DNA sequences see Supporting Table 3). DNA filament disassembly can be induced reversibly by UV illumination. Note that during the first illumination cycle the UV exposure was 3 min and 15 s for the others. **b** Porosity over time for the GUV shown in **a**. DNA filaments reassemble over the course of 30 min. Time points of UV illumination are indicated. Scale bar: 10 µm.

Supporting Figure 14: Dextran induces bundling of st DNA filaments in bulk and in GUVs

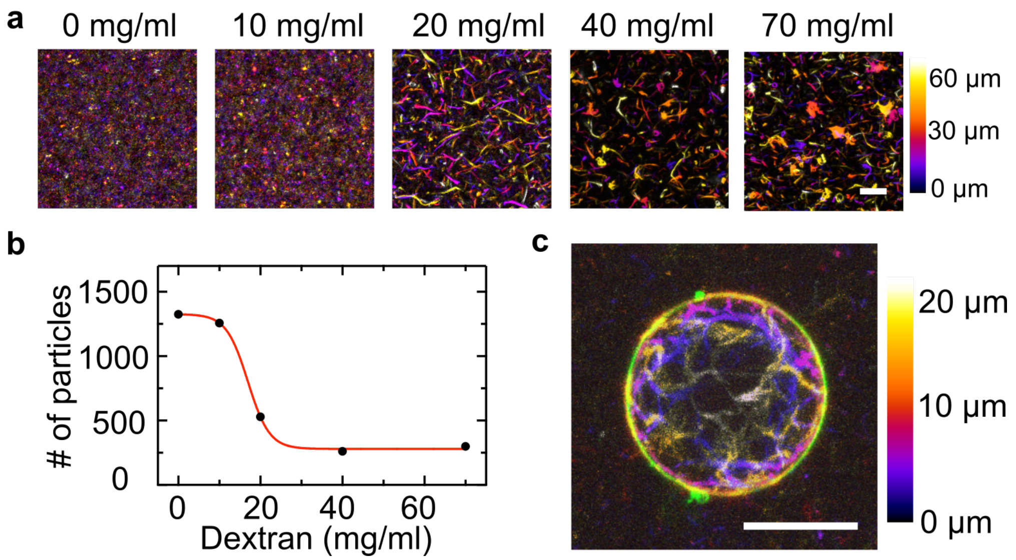

Figure 14: Dextran induces bundling of st DNA filaments in bulk and in GUVs. **a** Color-coded z-projections of st DNA filaments (λ_{ex}=561 nm) with varied amounts of 35 kDa dextran as indicated. Scale bar: 50 µm. **b** Number of detected filaments per image for varying dextran concentrations. The number of detected filaments corresponds to the degree of bundling as bundles are comprised of multiple st DNA filaments. **c** Overlay of the equatorial plane of a GUV (488 nm) and a color-coded z-projection of 500 nM st DNA filaments in presence of 20 mg/ml 35 kDa dextran. Scale bar: 10 µm.

Supporting Figure 15: Methylcellulose induces bundling of st DNA filaments in bulk

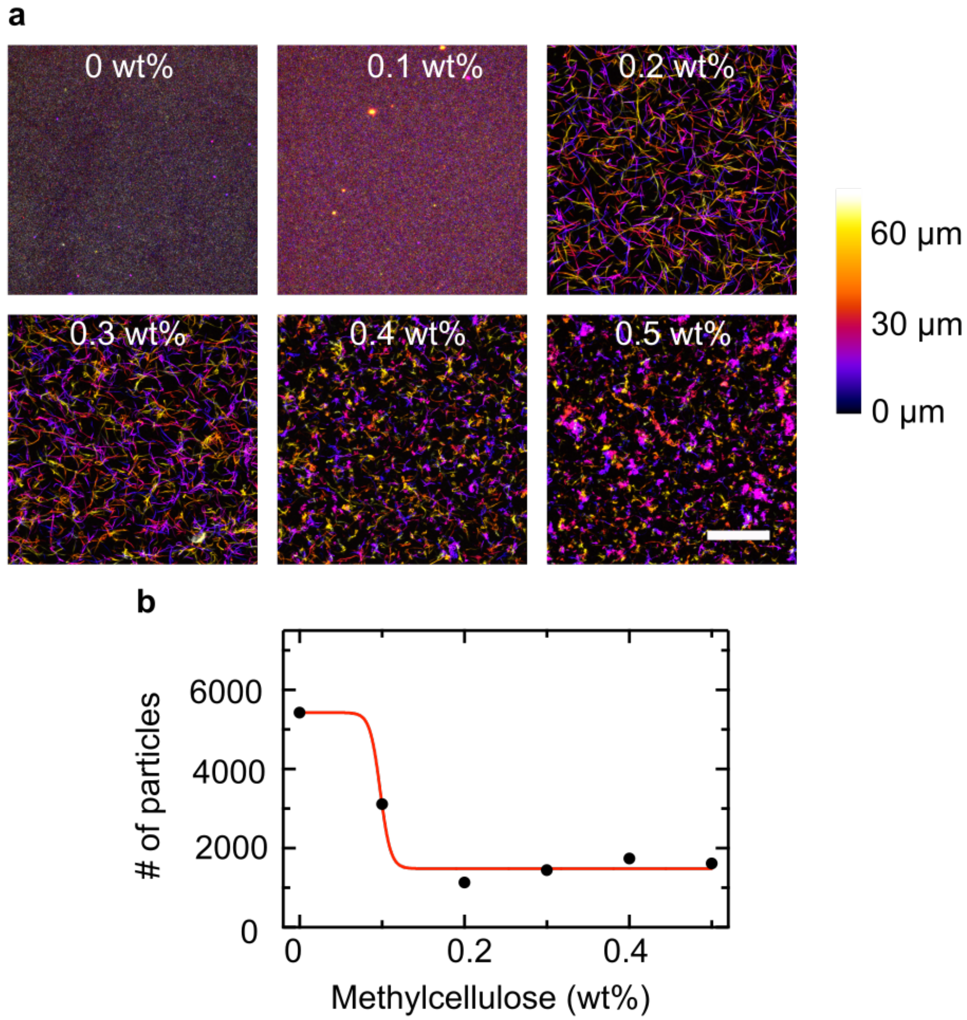

Figure 15: Methylcellulose (MC) induces bundling of st DNA filaments in bulk. **a** Colour-coded z-projections of st DNA filaments (λ_{ex}=561 nm) with varied amounts of MC as indicated. Scale bar: 50 µm. **b** Number of detected particles per image for different MC concentrations. The number of detected particles corresponds to the degree of bundling as bundles are comprised of multiple st DNA filaments. The number of particles was analyzed from confocal images using the Analyze Particle function in ImageJ.

Supporting Figure 16: Persistence length analysis

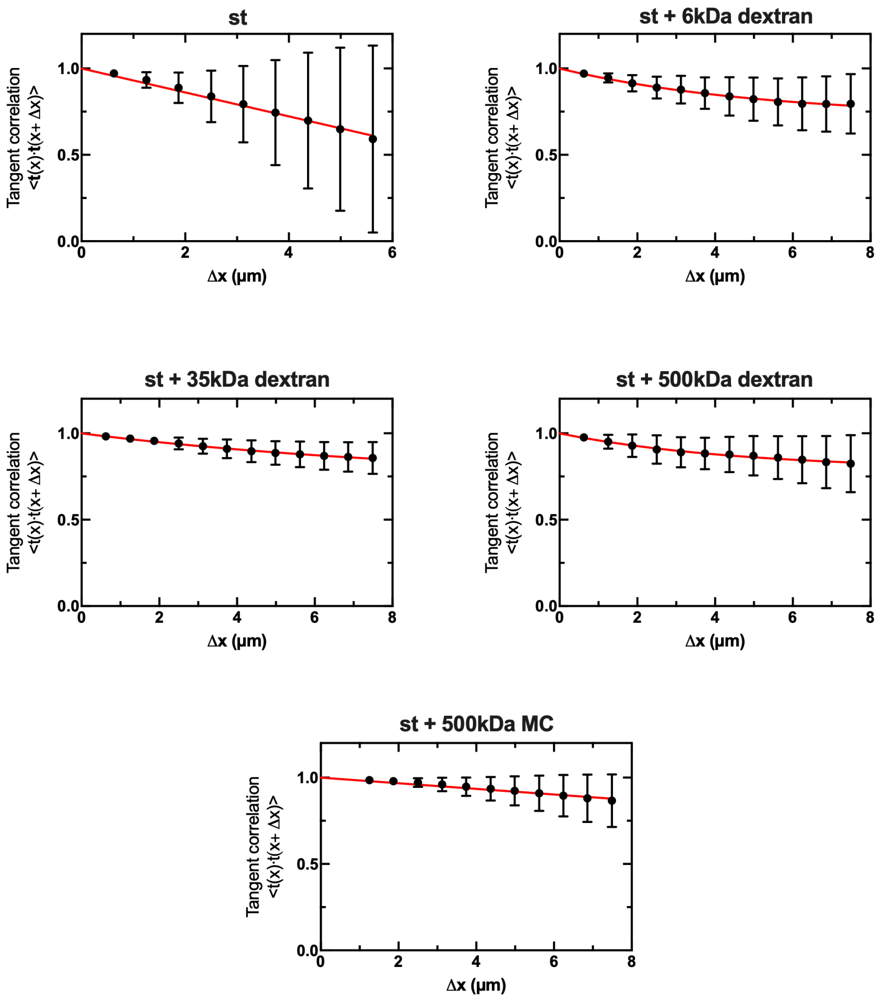

Figure 16: Persistence length analysis. Tangent correlation $\langle \hat{t}(x) \cdot \hat{t}(x + \Delta x) \rangle$ over the distance Δx between the tangents from all considered filaments for the conditions st (n = 15, l_p = 6.46 ± 0.26 µm), st +6 kDa dextran (n = 15, l_p = 12.81 ± 0.53 µm), st +35 kDa dextran (n = 11, l_p = 21.73 ± 0.59 µm), st +500 kDa dextran (n = 13, l_p = 17.09 ± 0.86 µm) and st + 500 kDa methylcellulose (n = 13, l_p = 26.75 ± 0.80 µm). The tangent correlation increases in presence of the molecular crowders.

Supporting Figure 17: Cryo electron micrographs of st DNA bundles formed by addition of dextran

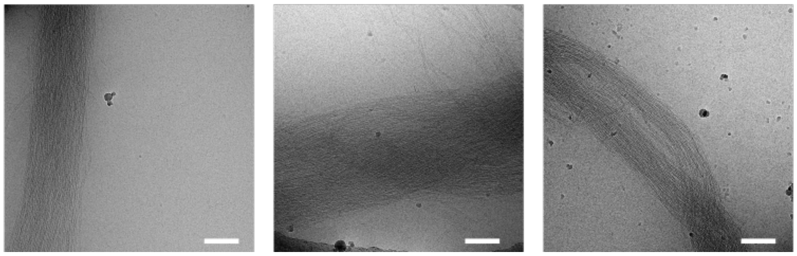

Figure 17: Cryo electron micrographs of st DNA bundles formed by addition of dextran to st DNA filaments. Scale bars: 200 nm.

Supporting Figure 18: TEM images of st DNA bundles formed by addition of methylcellulose

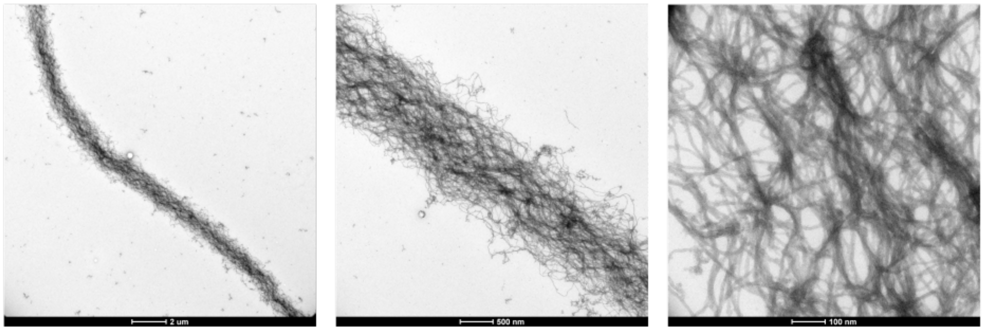

Figure 18: Transmission electron microscopy (TEM) images of DNA bundles formed by addition of methylcellulose (MC, 0.4 wt%) to st DNA filaments. 500 nM DNA filaments form long and thick bundles because MC acts as a crowding agent. Scale bars (from left to right): 4 µm, 1 µm and 200 nm.

Supporting Figure 19: st DNA bundles formed by addition of methylcellulose cannot be reconstituted into GUVs

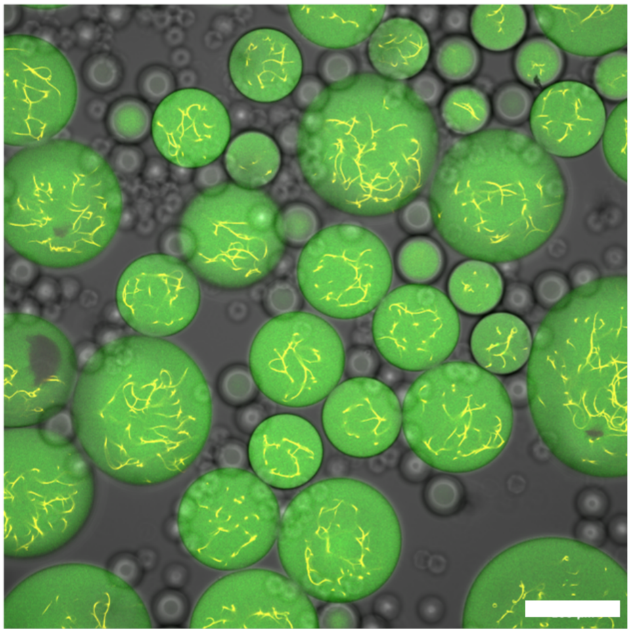

Figure 19: DNA bundles formed from st filaments by addition of methylcellulose (MC) cannot be reconstituted into GUVs using the droplet-stabilized GUV formation method.[2] Confocal image of st DNA bundles ($lambda_{ex}$ =561 nm inside water-in-oil droplets in presence of negatively charged fluorosurfactant Krytox and small unilamellar vesicles (SUVs, λ_{ex} = 488 nm). Even after up to three days, SUVs do not fuse with the droplet periphery likely due to the high viscosity of the MC-containing solution. Therefore, GUVs containing MC cannot be formed with the droplet-stabilized method. We thus used dextran as an alternative crowding agent. Scale bar: 100 µm.

Supporting Figure 20: Confocal overlay of bundled st DNA filaments form ring-like structures inside GUVs

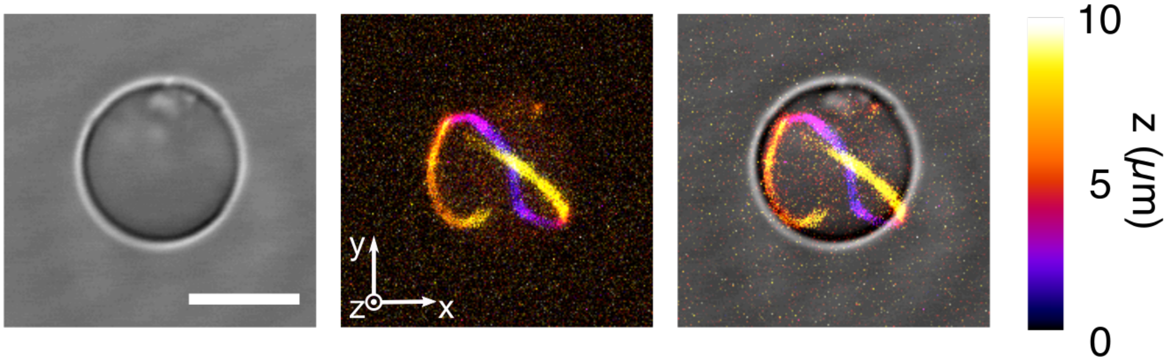

Figure 20: Color-coded confocal z-projection and corresponding brightfield image of 50 nM st filaments in presence of 20 mg/ml 35 kDa dextran inside a GUV. Scale bar: 5 μm.

Supporting Figure 21: Bundled st DNA filaments form ring-like structures inside GUVs

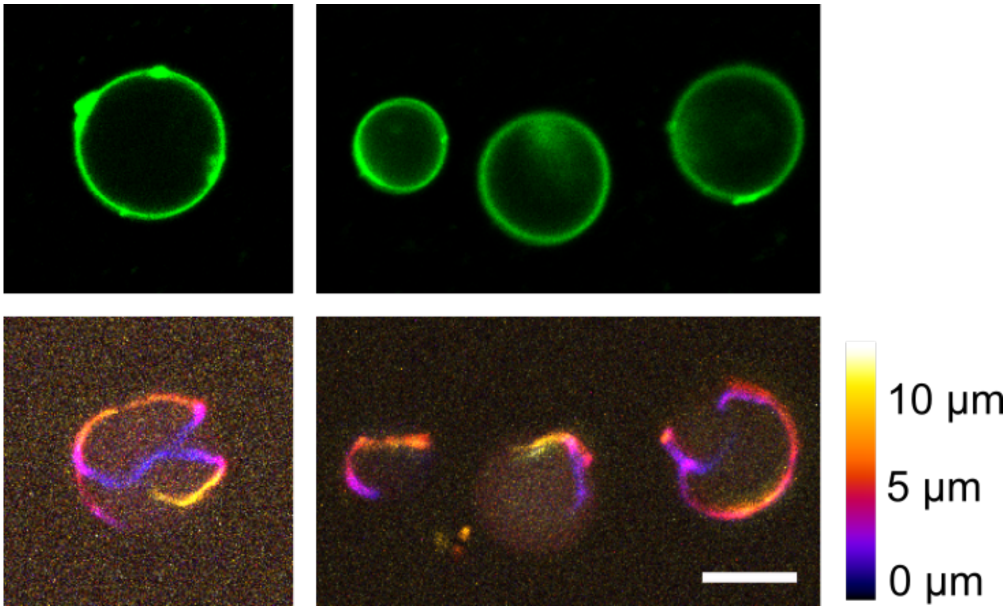

Figure 21: Bundled DNA filaments form ring-like structures inside GUVs. Exemplary confocal fluorescence images of GUVs (green, top row, λ_{ex}=488 nm) containing 200 nM DNA filaments and 30 mg/ml dextran as a bundling agent. DNA bundles (λ_{ex}=561 nm) are shown as colour-coded z-projections. After the formation of GUVs, the DNA filaments were re-annealed in the thermocycler. Scale bar: 10 µm.

Supporting Figure 22: Disassembly of bundled st-azo DNA filaments inside GUVs

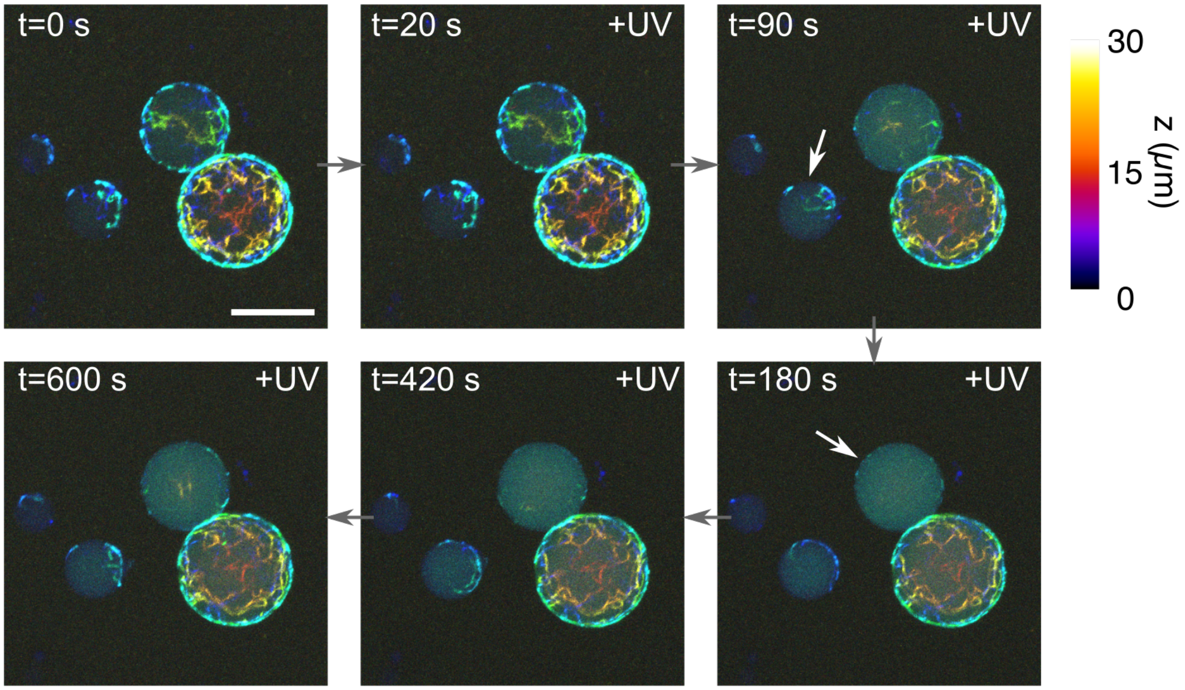

Figure 22: Color-coded z-projections of 500 nM st-azo DNA bundles (labelled with Cy3, λ_{ex} = 561 nm) within GUVs during continuous UV illumination over time. DNA bundles also form for st-azo DNA filaments. Filament disassembly requires longer illumination times than in absence of the molecular crowder (180 s vs. 20 s). Filament disassembly of bundled filaments only worked in smaller GUVs (indicated by a white arrow). This is likely due to the fact that DNA bundles in smaller GUVs consist of less DNA filaments due to their limited availability. Scale bar: 20 µm.

Supporting Figure 23: GUV formation efficiency increases in presence of cholesterol-tagged DNA filaments

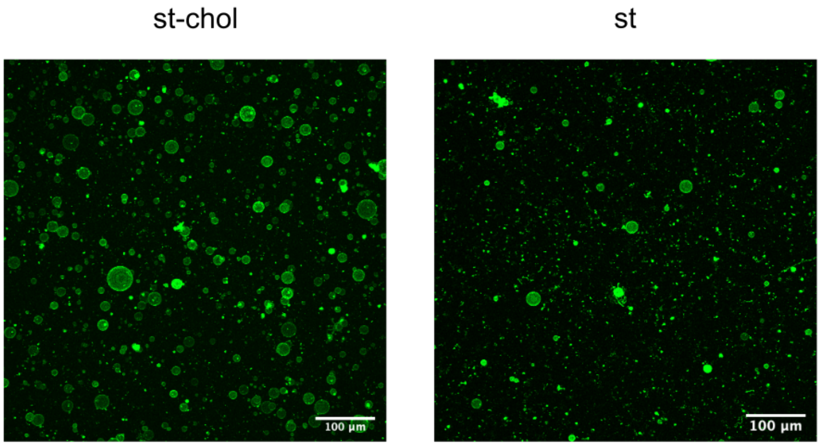

Figure 23: GUV formation efficiency increases in presence of membrane-bound st-chol DNA filaments. Confocal fluorescence z-projection of GUVs (λ_{ex}=488 nm) containing 1 µM st-chol or 500 nM st DNA filaments and 1 µM of cholesterol-tagged DNA. Each individual tile of the DNA filaments contains a complementary overhang for the cholesterol-tagged DNA (for DNA sequences see Supporting Table 3). The release efficiency increases more than five-fold from ≈ 1500 GUVs/µL for st filaments to ≈ 8000 GUVs/µL for st-chol filaments. Scale bar: 100 µm.

Supporting Figure 24: DNA filaments are recruited to the inner membrane of GUVs in the presence of cholesterol-tagged DNA

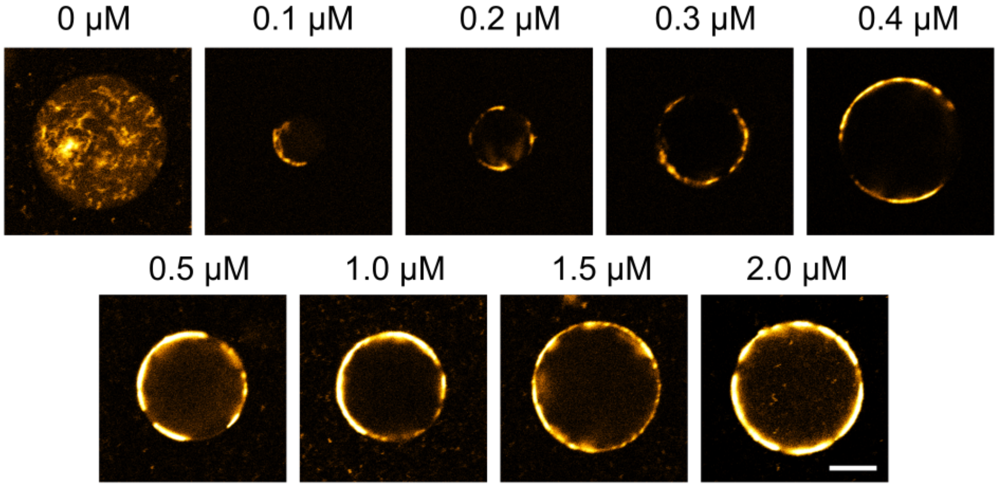

Figure 24: st DNA filaments are recruited to the inner membrane of GUVs in the presence of a cholesterol-tagged DNA which is complementary to a single-stranded overhang positioned on each individual st. Confocal fluorescence images of GUVs containing 500 nM st-chol DNA filaments (orange, λ_{ex} =561 nm) and varied amounts of cholesterol-tagged DNA. For DNA sequences see Supporting Table 3. Scale bar: 10 µm.

Supporting Figure 25: Membrane-bound DNA filaments suppress membrane fluctuations

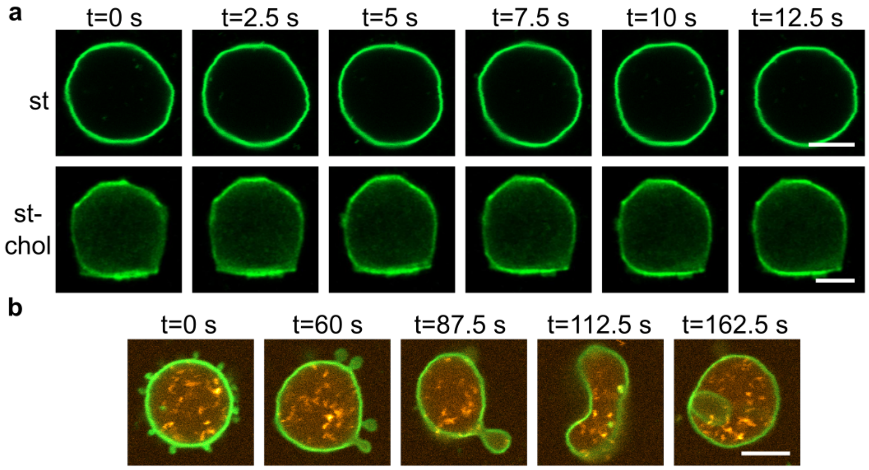

Figure 25: Membrane-bound DNA filaments suppress membrane fluctuations. **a** Confocal time series of deflated GUVs (green, 69% DOPC, 30% DOPG, 1% Atto488-DOPE, $\lambda_{ex} = 488\,\text{nm}$, $c_{\text{out}}/c_{\text{in}} = 600\,\text{mOsm}/300\,\text{mOsm} = 2$) containing normal st or membrane-bound st-chol DNA filaments over time. Scale bar: 10 µm and 5 µm, respectively. See Videos 6 and 7 for the entire time series. **b** Deflated st DNA filament-containing GUVs fluctuate and undergo shape transformations. Such dynamics cannot be observed for membrane-bound st-chol DNA filaments. Scale bar: 10 µm.

Supporting Tables

Supporting Table 1: DNA sequences for st DNA filaments

Table 1: DNA sequences from 5' to 3' for st DNA filaments, adapted from.[3]

Name	DNA sequence
S1	CTCAGTGGACAGCCGTTCTGGAGCGTTGGACGAAACT
S2	(Atto488-)TGGTATTGTCTGGTAGAGCACCACTGAGAGGTA
S3	CCAGAACGGCTGTGGCTAAACAGTAACCGAAGCA-<br>CCAACGCTGGTAAGTCTCCTTCTTATCT(-Cy3/Atto633)
S4	CAGACAGTTTCGTGGTCATCGTACCT
S5	CGATGACCTGCTTCGGTTACTGTTTAGCCTGCTCTAC

Supporting Table 2: DNA sequences for two-tile DNA filaments

Table 2: DNA sequences from 5' to 3' for two-tile DNA filaments.

Name	DNA sequence
RE1	CGTATTGGACATTTCCGTAGACCGACTGGACATCTTC
RE2	TCTACGGAAATGTGGCAGAATCAATCATAAGACACCAGTCGG
RE3	CCTCACCTTCACACCAATACGAGGTA
RE4	CAGACGAAGATGTGGTAGTGGAATGC
RE5	CCACTACCTGTCTTATGATTGATTCTGCCTGTGAAGG
SE1	CTCAGTGGACAGCCGTTCTGGAGCGTTGGACGAAACT
SE2	GTCTGGTAGAGCACCACTGAGGCATT
S3	CCAGAACGGCTGTGGCTAAACAGTAACCGAAGCA-<br>CCAACGCTGGTAAGTCTCCTTCTTATCT(-Cy3)
SE4	TGAGGAGTTTCGTGGTCATCGTACCT
SE5	CGATGACCTGCTTCGGTTACTGTTTAGCCTGCTCTAC

Supporting Table 3: DNA sequences for modified DNA filaments

Table 3: DNA sequences from 5' to 3' for modified DNA filaments. The symbol X represents the positioning of an azobenzene modification.

Name	DNA sequence
Invader	ACCAGACAATACCAATCCGC
S4-Azo	CAXGACAGTTTCGTGGTCATCGTACXCT
Chol-link	Chol-TTTAGATAAGAAGGAGACTTACC

Supporting Movies

Supporting Movie 1: Dynamics of st DNA filaments inside GUVs

Confocal fluorescence time series of 500 nM st DNA filaments (orange, λ_{ex}=561 nm) within GUVs (green, λ_{ex}=488 nm). DNA filaments are stably encapsulated and dynamic inside the GUV lumen. Scale bar: 10 µm.

Supporting Movie 2: Bundling of st DNA filaments with polyethylene glycol as molecular crowder

Confocal fluorescence time series of 500 nM st DNA filaments (λ_{ex}=561 nm) in presence of 5 wt% polyethyleneglycol (8000 Da, PEG-8k). DNA filaments condense into bundles within minutes due to molecular crowding induced by PEG-8k. Scale bar: 50 µm.

Supporting Movie 3: Formation of DNA cortex-like networks *via* bundling agents

Confocal fluorescence z-stack of 500 nM st DNA filaments (λ_{ex}=561 nm) in presence of 35 kDa dextran within a GUV. DNA filaments bundle and condense at the GUV periphery. Scale bar: 10 µm.

Supporting Movie 4: st-chol DNA filaments diffuse on SLBs

Confocal fluorescence time series of 5 nM st-chol DNA filaments (λ_{ex}=561 nm) on a supported lipid bilayer (SLB, λ_{ex}=488 nm). Filaments break and reform by diffusion on the lipid bilayer. Scale bar: 20 µm.

Supporting Movie 5: Formation of DNA cortex-like networks induced by cholesterol-tagged DNA-mediated linking

Confocal fluorescence z-stack of 500 nM st-chol DNA filaments (λ_{ex}=561 nm) within a GUV. DNA filaments condense at the GUV periphery due to recruitment by the cholesterol-tagged DNA, which is complementary to a single-stranded DNA overhang on the filaments. Scale bar: 10 µm.

Supporting Movie 6: GUVs are deformed by st-chol DNA filaments

Confocal fluorescence time series of 1 µM st-chol DNA filaments (λ_{ex}=561 nm) within GUVs (λ_{ex}=488 nm). DNA filaments deform the deflated GUV from within and suppress membrane fluctuations. Scale bar: 10 µm.

Supporting Movie 7: Deflated GUV in presence of st DNA filaments

Confocal fluorescence time series of 500 nM st DNA filaments (λ_{ex}=561 nm) within GUVs (λ_{ex}=488 nm). st DNA filaments do not deform deflated GUVs. Scale bar: 10 µm.

References

(1) Haller, B.; Göpfrich, K.; Schröter, M.; Janiesch, J.-W.; Platzman, I.; Spatz, J. P. Charge-Controlled Microfluidic Formation of Lipid-Based Single- and Multicompartment Systems. *Lab on a Chip* **2018**, *18*, 2665–2674.

(2) Weiss, M.; Frohnmayer, J. P.; Benk, L. T.; Haller, B.; Janiesch, J.-W.; Heitkamp, T.; Börsch, M.; Lira, R. B.; Dimova, R.; Lipowsky, R.; Bodenschatz, E.; Baret, J.-C.; Vidakovic-Koch, T.; Sundmacher, K.; Platzman, I.; Spatz, J. P. Sequential Bottom-Up Assembly of Mechanically Stabilized Synthetic Cells by Microfluidics. *Nature Materials* **2017**, *17*, 89–96.

(3) Rothemund, P. W. K.; Ekani-Nkodo, A.; Papadakis, N.; Kumar, A.; Fygenson, D. K.; Winfree, E. Design and Characterization of Programmable DNA Nanotubes. *Journal of the American Chemical Society* **2004**, *126*, 16344–16352.
